# Inflammatory changes in the choroid plexus following subarachnoid hemorrhage: the role of innate immune receptors and inflammatory molecules

**DOI:** 10.3389/fncel.2024.1525415

**Published:** 2025-01-07

**Authors:** Peter Solár, Václav Brázda, Martin Bareš, Alemeh Zamani, Parisa EmamiAref, Andrea Joukal, Lucie Kubíčková, Erik Kročka, Klaudia Hašanová, Marek Joukal

**Affiliations:** ^1^Department of Anatomy, Faculty of Medicine, Masaryk University, Brno, Czechia; ^2^Department of Neurosurgery, St. Anne’s University Hospital, and Faculty of Medicine, Masaryk University, Brno, Czechia; ^3^Institute of Biophysics, Academy of Sciences of the Czech Republic, Brno, Czechia

**Keywords:** subarachnoid hemorrhage, stroke, choroid plexus, blood-cerebrospinal fluid barrier, neuroinflammation, hydrocephalus

## Abstract

**Introduction:**

The choroid plexus is located in the cerebral ventricles. It consists of a stromal core and a single layer of cuboidal epithelial cells that forms the blood-cerebrospinal barrier. The main function of the choroid plexus is to produce cerebrospinal fluid. Subarachnoid hemorrhage due to aneurysm rupture is a devastating type of hemorrhagic stroke. Following subarachnoid hemorrhage, blood and the blood degradation products that disperse into the cerebrospinal fluid come in direct contact with choroid plexus epithelial cells. The aim of the current study was to elucidate the pathophysiological cascades responsible for the inflammatory reaction that is seen in the choroid plexus following subarachnoid hemorrhage.

**Methods:**

Subarachnoid hemorrhage was induced in rats by injecting non-heparinized autologous blood to the cisterna magna. Increased intracranial pressure following subarachnoid hemorrhage was modeled by using artificial cerebrospinal fluid instead of blood. Subarachnoid hemorrhage and artificial cerebrospinal fluid animals were left to survive for 1, 3, 7 and 14 days. Immunohistochemical staining of TLR4, TLR9, FPR2, CCL2, TNFα, IL-1β, CCR2 and CX3CR1 was performed on the cryostat sections of choroid plexus tissue. The level of TLR4, TLR9, FPR2, CCL2, TNFα, IL-1β was detected by measuring immunofluorescence intensity in randomly selected epithelial cells. The number of CCR2 and CX3CR1 positive cells per choroid plexus area was manually counted. Immunohistochemical changes were confirmed by Western blot analyses.

**Results:**

Immunohistochemical methods and Western blot showed increased levels of TLR9 and a slight increase in TLR4 and FRP2 following both subarachnoid hemorrhage as well as the application of artificial cerebrospinal fluid over time, although the individual periods were different. The levels of TNFα and IL-1β increased, while CCL2 level decreased slightly. Accumulation of macrophages positive for CCR2 and CX3CR1 was found in all periods after subarachnoid hemorrhage as well as after the application of artificial cerebrospinal fluid.

**Discussion:**

Our results suggest that the inflammation develops in the choroid plexus and blood-cerebrospinal fluid barrier in response to blood components as well as acutely increased intracranial pressure following subarachnoid hemorrhage. These pro-inflammatory changes include accumulation in the choroid plexus of pro-inflammatory cytokines, innate immune receptors, and monocyte-derived macrophages.

## 1 Introduction

Subarachnoid hemorrhage (SAH) is a type of hemorrhagic stroke that accounts approximately for 5% of all strokes. The incidence of SAH depends on the geographical location and varies from 0.5 to 28 per 100,000 person-years (Etminan et al., 2019). SAH is a devastating cerebrovascular disease and is a significant cause of morbidity and mortality all over the world. More than 80% SAH cases are due to the rupture of a cerebral aneurysm (Connolly et al., 2012). Intracranial pressure increases rapidly upon aneurysm rupture and blood fills the subarachnoid space. Along with blood components, this sudden rise in intracranial pressure (ICP) triggers the initial pathophysiological cascades resulting in early brain injury (EBI) and the later complex reactions known as delayed cerebral ischemia (DCI) (van Lieshout et al., 2018). One of the sites where SAH-induced changes occur is the choroid plexus (CP) (Solár et al., 2020b,a,c). The CP is found in the brain ventricles. It forms the blood-cerebrospinal fluid barrier (B-CSF barrier) and is responsible for production of cerebrospinal fluid (CSF). The CP consists of a highly vascularized stroma containing fenestrated capillaries covered with a single layer of cuboidal epithelial cells. The epithelial cells are connected by tight junction (TJ) proteins that form the main component of the B-CSF barrier. The production of CSF by the cuboidal epithelial cells is controlled by the B-CSF barrier that maintains homeostasis of the central nervous system (CNS) through the selective transport of ions and molecules. Epiplexus cells, also called as Kolmer cells (KC), exhibit phagocytic activity, adhere to the ventricular side of the cuboidal epithelial cells and are considered immune cells direct contact with CSF (Wolburg and Paulus, 2010; Solár et al., 2020c). Following SAH, we have previously observed increased numbers of KC with the activated (ED1+) immunophenotype, along with increased numbers of resident (ED2+) macrophages, CCR7+ and CD206+ cells and MHC II+ antigen presenting cells. Interestingly, similar changes were also found (but to a lesser extent) following the application of artificial cerebrospinal fluid (ACSF) indicating that increased intracranial pressure can also contribute to the observed changes (Solár et al., 2020b). Accumulation of immune cells with increased hemoxygenase-1 (HO-1) amounts predominantly in the epiplexus position indicates that they have a role to play in hemoglobin degradation. Increased levels of HO-1 in activated and resident macrophages in the epiplexus position as well as biliverdin-reductase (BVR) in epithelial cells may also contribute to the restoration of CNS homeostasis (Solár et al., 2020a). On the other hand, accumulation of immune cells of different phenotypes in the CP may contribute to a pro-inflammatory response resulting in hypersecretion of CSF and thus in the development of post-hemorrhagic hydrocephalus, which is one of the major complications after SAH (Karimy et al., 2017; Wan et al., 2019). However, the precise source of the trigger and its mechanism underlying immune cell accumulation in the CP following SAH remains a mystery.

Toll like receptors (TLRs), a class of pattern recognition receptors (PRRs), are transmembrane and intracellular molecules containing domains comprised largely of leucine-rich repeats and intracellular domains that activate signaling cascades (Akira et al., 2006). TLRs are one of the main effectors in the innate immune system (Iwasaki and Medzhitov, 2004). Activation of TLRs either by various pathogen-associated molecular patterns (PAMPs) or endogenous molecules from damaged tissue [called damage-associated molecular patterns (DAMPs)] leads to the increased expression of inflammatory molecules involved in chemotaxis and phagocytosis (Wu et al., 2009; Piccinini and Midwood, 2010). TLRs located on the membranes of CP epithelial cells have an important role in CNS immunity. Moreover, their activation may lead to cytoskeleton restructuring and the downregulation of junctional proteins of the B-CSF barrier resulting in hypersecretion of CSF. Alteration of the B-CSF barrier along with the activation of proinflammatory genes may play an important role in attracting circulating monocytes and their transition to the CP inducing various diseases (Stridh et al., 2013; Thompson et al., 2022). The aim of our present work was to assess changes of DAMP receptors in response to experimentally induced SAH or increased intracranial pressure induced by the application of ACSF. We also focused on some of the proinflammatory signaling cascades triggered by SAH or increased intracranial pressure in the CP that could influence or modulate leukocyte chemotaxis and accumulation.

## 2 Materials and methods

All experimental protocols, including animal use, care protocols and all operation procedures were approved by the Ethical Committee of Masaryk University, Brno, and the Departmental Committee of the Ministry of Education, Youth and Sports, Czechia (Approval No.: MŠMT 21101/2016–3). We used 135 adult male rats (8 weeks old Wistar rats weighing 200–250 gr; Animal Breeding Facility, Masaryk University, Czechia) in our study (Table 1).

**Table 1:** Table showing distribution and numbers of animals in individual groups.

	Immunohistochemical staining				Western blot			
	1D (n)	3D (n)	7D (n)	14D (n)	1D (n)	3D (n)	7D (n)	14D (n)
SAH	10	10	10	10	5	5	5	5
ACSF	10	10	10	10	5	5	5	5
Naive	10				5			

### 2.1 Experimental models of subarachnoid hemorrhage

There are three main models of SAH described in the literature: prechiasmatic, perforating and blood application into the cisterna magna. In the prechiasmatic model, distribution of blood in the subarachnoid space is similar to that after the rupture of an aneurysm on the artery of the circle of Willis and thus is similar to the clinical course of SAH. The disadvantage of this model is that it is time-consuming, needs a stereotactic frame and there is no vascular damage (Prunell et al., 2002). Vascular damage occurs in the perforation model, which mimics the rupture of an aneurysm. The basis of the perforation model is in most cases a rupture of the anterior cerebral artery (Veelken et al., 1995). The advantage of the perforation model is vascular damage, thus modeling the real mechanism of arterial aneurysm rupture. However, a big disadvantage is the large variability in the volume of blood spilled into the subarachnoid space and high mortality of experimental animals. This model is often used to evaluate acute changes following SAH.

In our study we used the animal model of SAH using the application of autologous arterial blood to the cisterna magna (Solomon et al., 1985). Generally, this model involves exposing the atlanto-occipital membrane and the slow injection of autologous blood through this membrane into the cisterna magna. Similar to the prechiasmatic bleeding model, the main disadvantage is that there is no vascular damage. On the other hand, the SAH model with blood application to the cisterna magna is frequently used for its relative simplicity, repeatability and constant volume of injected blood. In contrast to other models, where the reduction of cerebral blood flow was at least 60 min following the induction of SAH, using the cisterna magna model, blood flow is restored 15 min after the application of blood into the cisterna magna (Prunell et al., 2003). Studies dealing with acute changes after SAH tend to use models with a longer-lasting reduction in cerebral blood flow compared with cisterna magna blood application (Jackowski et al., 1990; Bederson et al., 1998; Prunell et al., 2003). Therefore, it is reasonable to assume that the SAH model with the application of blood into the cisterna magna is more suitable for studying the changes induced by blood and blood degradation products that occur rather late after SAH (Prunell et al., 2003).

### 2.2 Cisterna magna model of subarachnoid hemorrhage

Animals were anesthetized with a mixture of 5% ketamine (100 mg/kg) and 2% xylazine (10 mg/kg) administrated intraperitoneally. To induce SAH we applied non-heparinized autologous arterial blood into the cisterna magna following the method originally published by Solomon et al., 1985 and later modified by other authors, including our group (d’Avella et al., 1996; Matz et al., 1996; Solár et al., 2020b,a). We first performed cannulation of the caudal artery in animals belonging to the SAH group. A midline suboccipital incision was made to expose the atlas, occipital bone, and atlanto-occipital membrane. Subsequently, the animals were positioned in a stereotaxic apparatus (Kopf Instruments, Tujunga, CA, United States). Then, the atlanto-occipital membrane was cleared of connective tissue and rendered clearly visible. A syringe with a 30G needle was placed on the manipulating arm of the stereotaxic apparatus tilted at 60 degrees from the horizontal plane. Using an operating microscope under high magnification, 300 μL of freshly obtained non-heparinized autologous arterial blood was injected into the cisterna magna within 60 s to the animals in the SAH group (d’Avella et al., 1996).

### 2.3 Induction of increased intracranial pressure

The animals in the ACSF group were injected with 300 μL of ACSF into the cisterna magna within 60 s. ACSF consisted of 130 mM NaCl, 3.0 mM KCl, 1.2 mM NaH2PO4, 20 mM NaHCO3, 1.3 mM MgCl2, 2.4 mM CaCl2, and 10 mM Glucose (Hylden and Wilcox, 1980). The ACSF for our experiment was prepared fresh and the pH was set to 7.40 ± 0.05. After administration of blood or ACSF the needle was slowly withdrawn over 2 min. To prevent blood or ACSF leakage, the puncture site in the atlanto-occipital membrane was closed with a gelatin sponge. The muscles were sutured using 2.0 absorbable suture and the skin was closed using 4–0 silk suture. The animals exposed to blood (SAH group) or ACSF (ACSF group) were left to survive for 1, 3, 7 and 14 days (n = 10 SAH; n = 10 ACSF for each time point). The rats in SAH and ACSF groups along with naïve animals (n = 10) were sacrificed by CO2 inhalation. Thereafter, 500 mL heparinized (1,000 units/500 mL) phosphate–buffered saline (PBS; pH 7.4) was used for transcranial perfusion followed by 500 mL of Zamboni’s fixative (Zamboni, 1967). The brains were removed immediately and assessed for appropriate changes. Animals where SAH was induced (SAH group) with the presence of blood clot in the subarachnoid cisterns and the basal surface of the brain were included. For the ACSF group, only animals without any blood clot in the subarachnoid space were included.

Included brains were kept in Zamboni’s fixative for 72 h, then rinsed in 10% sucrose and embedded in Tissue-Tek OCT compound (Miles; Elkhart, IN, United States). Serial coronal cryostat sections (20 μm) were cut (Leica 1800 cryostat; Leica Microsystem, Wetzlar, Germany) and mounted onto chrome-alum covered microscopic slides.

### 2.4 Immunohistochemical staining

To detect pro-inflammatory receptors and molecules, the brain sections of naive, SAH and ACSF groups were immunostained under identical conditions with anti-TLR4, anti-TLR9, anti-FPR2, anti-CCR2, anti-CCL2, anti-CX3CR1, anti-IL-1β and anti-TNFα antibodies (Table 2). Sections were washed with PBS containing 0.3% bovine serum albumin and 0.1% Tween-20, treated with 3% normal serum for 30 min and then incubated with the primary antibody at room temperature. Primary antibody sources and incubation conditions are shown in Table 2. Affinity purified Cy5-conjugated donkey anti-rabbit (Jackson, 1:100) was applied at room temperature for 90 min. Control sections were incubated in parallel omitting the primary antibodies. Immunostained sections were rinsed, stained with Hoechst 33342 (Sigma, St. Louis, MO, United States) to locate cell nuclei, and mounted in Vectashield aqueous mounting medium (Vector Laboratories, Burlingame, CA, United States). Immunofluorescence was observed and analyzed using a Nikon Eclipse NI-E epifluorescence microscope, equipped with a Nikon DS-Ri1 camera (Nikon, Prague, Czechia).

**Table 2:** List of primary antibodies used, their dilutions, incubation times and suppliers.

Name	Type of antibody	Dilution	Incubation time	Supplier
TLR4	Rabbit polyclonal	1:100	24 h	Santa Cruz
TLR9	Rabbit polyclonal	1:500	Overnight	Acris
FPR2	Rabbit polyclonal	1:100	Overnight	Novusbio
TNFα	Rabbit polyclonal	1:500	Overnight	Abcam
IL-1β	Rabbit polyclonal	1:100	Overnight	LSBio
CCL2	Rabbit polyclonal	1:100	Overnight	MyBioSource
CCR2	Rabbit polyclonal	1:100	Overnight	MyBioSource
CX3CR1	Rabbit polyclonal	1:200	Overnight	Abcam

### 2.5 Western blot analysis

**Figure 1:**
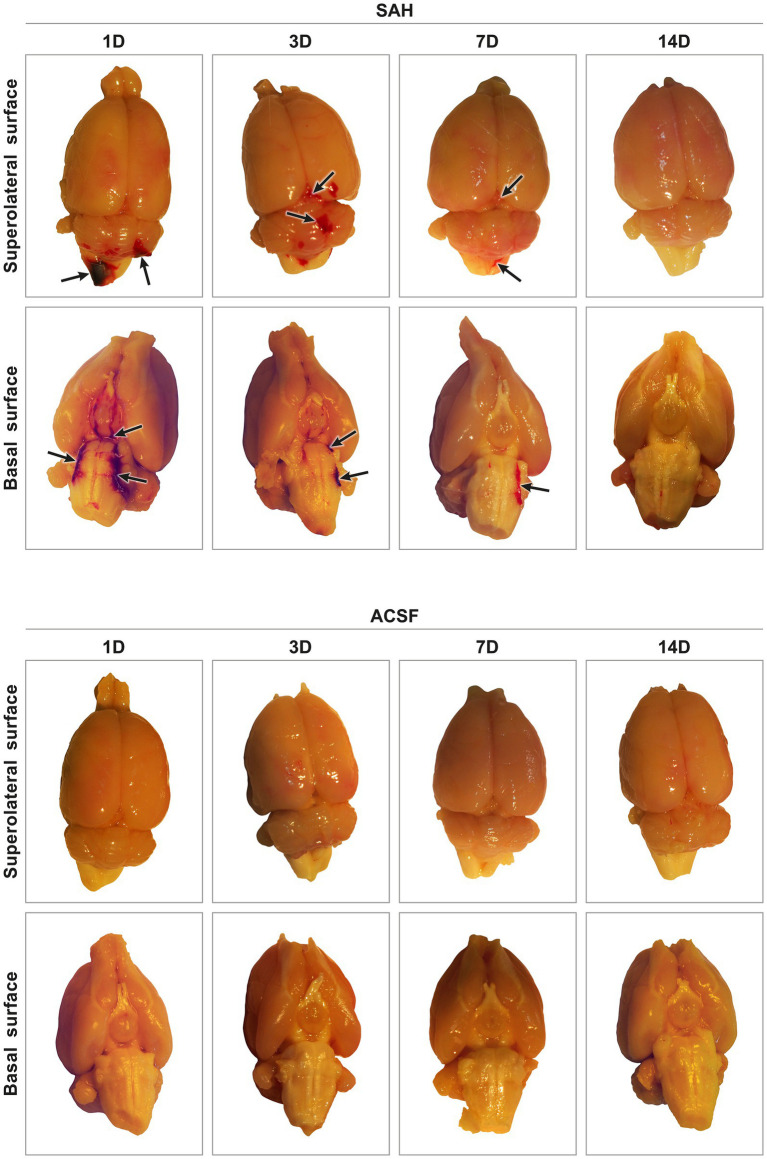
Representative pictures of harvested brain tissue from animals after SAH induction or ACSF application. In experimental animals after SAH induction, blood was found in the subarachnoid spaces on the basal surface, as well as on the superolateral surface of the brain. The highest amount of blood in the subarachnoid space was found after 1 day and the least amount 14 days after introduction into the cisterna magna. ACSF application into the cisterna magna did not result in accumulation of blood in the subarachnoid space. Arrows point out no blood clot in the subarachnoid space of the brain.

Western blot analysis was used to verify changes in the levels of selected proteins in the choroid plexus obtained by measuring the intensity of immunofluorescence. Rats in the naive group (n = 5), the SAH group, and the ACSF group were kept alive 1 (*n* = 5 SAH; *n* = 5 ACSF), 3 (*n* = 5 SAH; *n* = 5 ACSF), 7 and 14 days (*n* = 5 SAH; *n* = 5 ACSF) and sacrificed by CO2 inhalation. Choroid plexus of both lateral ventricles was collected under aseptic conditions, immersed in protease inhibitor and phosphatase inhibitor cocktails (Roche, Germany), frozen in liquid nitrogen and stored at −80°C until analysis. Tissue samples from each group of animals were collected and homogenized in PBS with 0.1% Triton X-100 and protease inhibitors (LaRoche, Switzerland) and centrifuged at 10,000 × g for 5 min at 4°C. Proteins were further separated by SDS—polyacrylamide gel electrophoresis as previously published (Brazda et al., 2006). The cells were blocked with 1% BSA in PBST (3.2 mM Na2HPO4, 0.5 mM KH2PO4, 1.3 mM KCl, 135 mM NaCl, 0.05% Tween 20, and pH 7.4) for 1 h at room temperature and incubated with specific antibodies at recommended dilutions: TLR4 (1: 500), TLR9 (1: 500), FPR2 (1:1000), CCL2 (1:1000), CCR2 (1:100), CX3CR1 (1:500), IL-1β (1:500), TNFα (1: 1000), overnight. The cells were further washed in PBST and incubated with peroxidase-conjugated anti-rabbit or mouse IgG (Sigma, 1:1000) at room temperature for 1 h. As a control protein was used vinculin. The ECL detection kit (Amersham) was used to visualize the protein bands in the LAS-3000 chemiluminometer (Supplementary Figure S1: Figure 1).

### 2.6 Image analysis

Digital immunofluorescence images of CP (magnification 200×) were obtained from at least 10 sections selected at 60 μm intervals from serial brain sections which included the CP of lateral brain ventricles. In all groups of animals each image of the CP was acquired using identical settings for the camera, optics, lamps, and under the same exposure conditions. Images of the CP were stored in TIFF format. Investigators blinded to the animal groups analyzed the images in the NIS-Elements AR Analysis software (version 4.20.00, Nikon, Prague, Czechia) according to our previously published protocols (Dubový et al., 2002, 2013).

### 2.7 Analysis of intensity of immunofluorescence

The intensity of TLR4, TLR9, FPR2, CCL2, TNFα, and IL-1*β* immunofluorescence was measured in clearly demarcated CP epithelial cells. A binary mask was created manually to detect cell boundaries and exclude nuclei. At least 100 randomly selected epithelial cells from each animal group were examined for immunofluorescence intensity after subtraction of background. The analysis was performed in a randomized fashion by two investigators who were blinded to the group of animals. The intensity of TLR4, TLR9, FPR2, CCL2, TNFα, and IL-1 β immunofluorescence in CP epithelial cells were expressed as means ± SD.

### 2.8 Analysis of the number of immunopositive cells

The number of CCR2 and CXCR1 positive Kolmer cells was counted per unit area of the CP. Firstly, the CP area was manually determined in the images and its extent was measured; the images were edited when needed. CCR2 and CXCR1 positive Kolmer cells were correlated with the position of cell nuclei (identified by Hoechst staining), and manually counted in the defined CP area. The analysis was performed in a randomized fashion by two investigators who were blinded to the group of animals. The number of CCR2 and CXCR1 positive Kolmer cells for an area of 1 mm^2^ of the CP is shown as means ± SD.

### 2.9 Statistical analysis

The data obtained from immunoquantification from naive, ACSF and SAH groups were compared using multiple comparisons of mean ranks for all groups (Kruskal–Wallis with Bonferroni *post hoc* test; *p* < 0.05; *p* < 0.01) in STATISTICA 13.2 software (StatSoft, Tulsa, OK, United States).

## 3 Results

All animals that underwent induction of SAH by applying blood into the cisterna magna or injection of ACSF survived the experiments. The presence of blood in the subarachnoid space (SAH group) indicated the successful application of blood in all animals. The most obvious blood clots were found in animals 1 and 3 days following SAH induction. Blood clots were found mainly on the ventral surface of the brain stem, in the basal cisterns. In the later stages of SAH, blood clots had been partially removed at 7 days and completely removed 14 days after blood application (Figure 1). In animals after application of ACSF, no blood clots or brain damage were found in the subarachnoid space.

### 3.1 Immunohistochemical staining of the pattern recognition receptors TLR4, TLR9, and FPR2 in the choroid plexus

**Figure 2:**
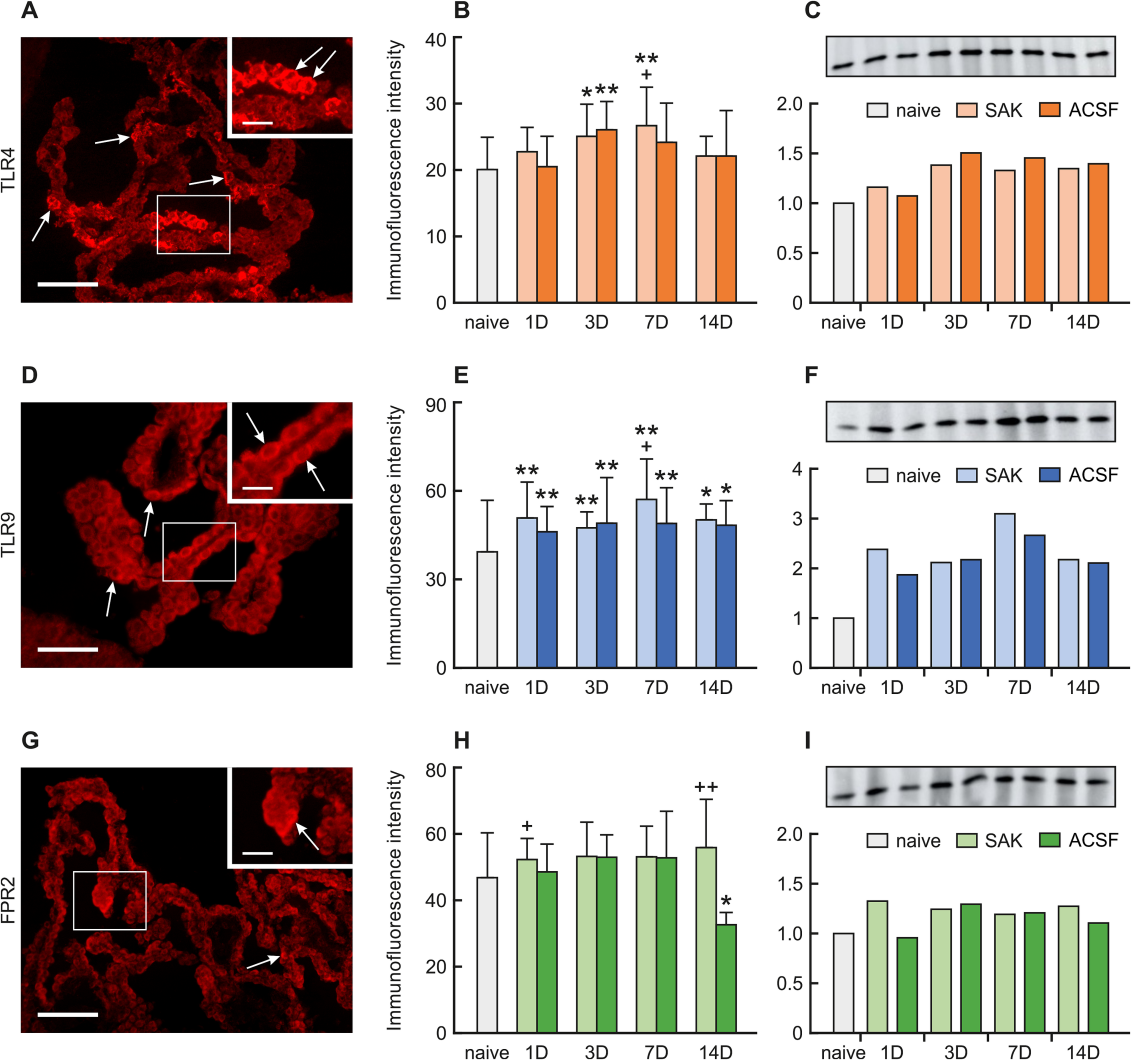
Representative pictures showing TLR4, TLR9 and FPR2 immunostaining in the CP after SAH induction. Arrows show TLR4, **(A)**, TLR9 **(D)**, and FPR2 **(G)** immunopositive CP epithelial cells. Insets at top right show higher magnifications of the regions indicated by the box in the main picture. Scale bars—main images 80 μm; insets 20 μm. Graphs show mean intensity (±SD) of TLR4 **(B)**, TLR9 **(E)**, and FPR2 **(H)** immunofluorescence in CP epithelial cells. Representative Western blot analysis comparing the levels of TLR4 **(C)**, TLR9 **(F)**, and FPR2 **(I)** in the CP from different experimental groups. *Significant difference compared to CP from naïve rats (*p < 0.05; **p < 0.01). +Significant difference compared to CP from ACSF rats (^+^*p* < 0.05; ^++^*p* < 0.01 *p* < 0.01).

A positive reaction to TLR4 was found mainly on the ventricular surface of the CP epithelial cells. Immunohistochemical staining showed a significantly increased TLR4 intensity 3 (*p* < 0.05) and 7 days (*p* < 0.01) following SAH induction as well as 3 days after ACSF application (p < 0.01) when compared to naïve animals. TLR4 immunofluorescence intensity was significantly increased 7 days after SAH induction when compared with ACSF animals at the same time point (< 0.05). The immunopositivity peaked at 7 days after SAH induction of and 3 days after ACSF application, and declined thereafter (Figures 2A,B; Supplementary Figures 2SA–I).

A positive reaction to TRL9 was found intracellularly, mainly on the apical side of the CP epithelial cells. TLR9 immunoflourescence intensity was significantly increased 1 (*p* < 0.01), 3 (*p* < 0.01), 7 (*p* < 0.01) and 14 days (*p* < 0.05) following SAH induction of as well as after ACSF application when compared to naïve animals. Immunofluorescence intensity was highest in animals 7 days after SAH induction of with a subsequent decline. Moreover, at this time point, the intensity of TLR9 was significantly higher (< 0.05) in the SAH group compared with ACSF animals. As for the ACSF group, the highest values of TLR9 were obtained from animals 3 and 7 days after ACSF application with a slight decrease over the following days (Figures 2D,E; Supplementary Figures 3SA–I).

A positive reaction to FPR2 was found mainly on the apical surface of CP epithelial cells. A non-significant increase in immunofluorescence was found in all periods after SAH induction. Increased FPR2 immunofluorescence was observed 1 (*p* < 0.05) and 14 days (*p* < 0.01) following SAH when compared to ACSF animals at the same time points. The intensity of the immune reaction showed a non-significant increase in the ACSF group 1, 3 and 7 days and significantly decreased 14 days after operation (*p* < 0.05) (Figures 2G,H; Supplementary Figures 4SA–I).

### 3.2 Immunohistochemical staining of TNFα, CCL2, and IL-1β in the choroid plexus

A positive reaction to TNFα, CCL2, and IL-1β was mainly found in the cuboidal epithelial cells of the CP in all groups of animals.

**Figure 3:**
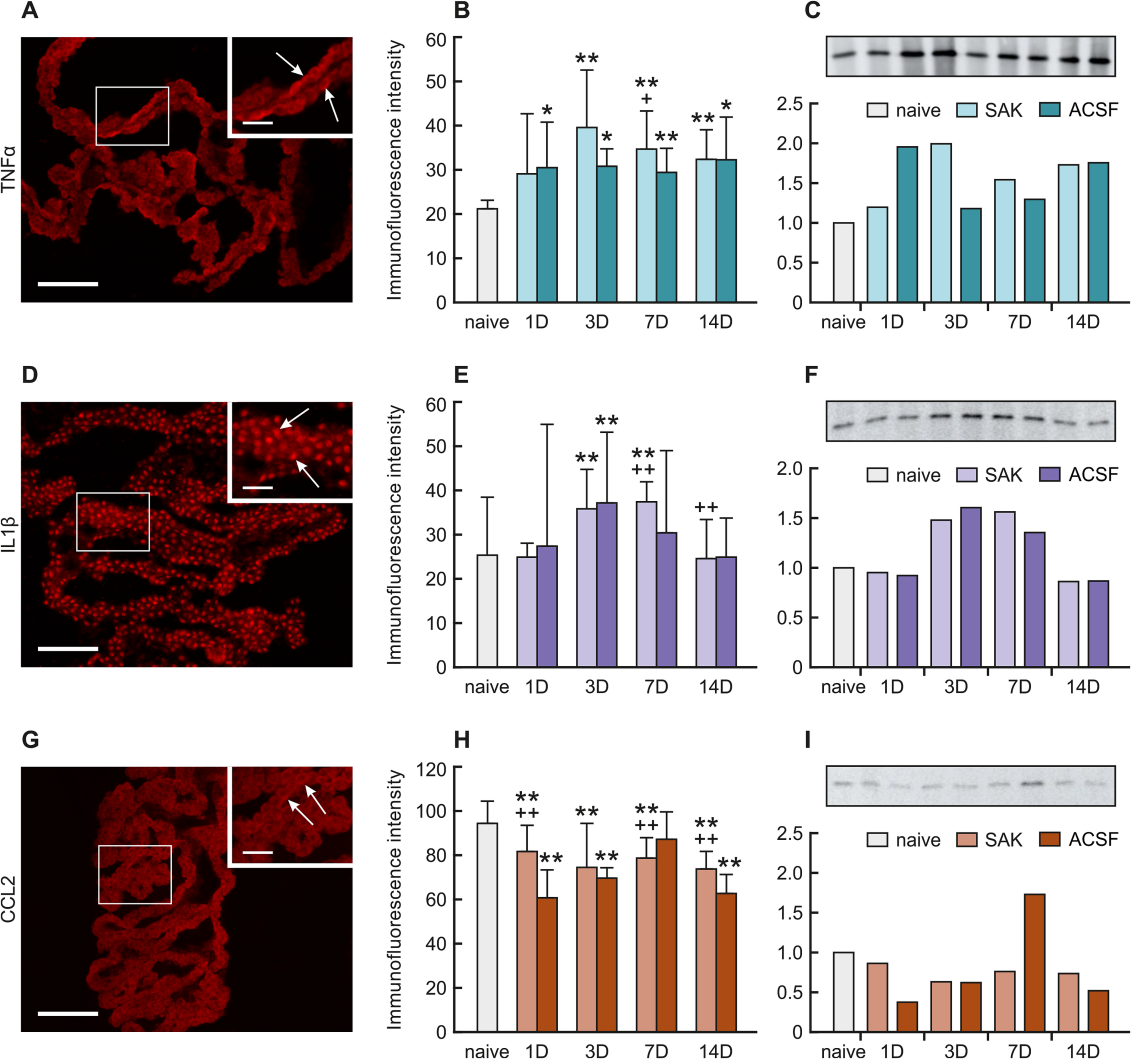
Representative pictures showing TNFα, IL-1β and CCL2 immunostaining in the CP after SAH induction of. Arrows show TNFα, **(A)**, IL-1β **(D)**, and CCL2 **(G)** immunopositive CP epithelial cells. Insets at top right show higher magnifications of the regions indicated by the box in the main picture. Scale bars—main images 80 μm; insets 20 μm. Graphs show mean intensity (±SD) of TNFα **(B)**, IL-1β **(E)**, and CCL2 **(H)** immunofluorescence in CP epithelial cells. Representative Western blot analysis comparing the levels of TNFα **(C)**, IL-1β **(F)**, and CCL2 **(I)** in the CP from different experimental groups. *Significant difference compared to CP from naïve rats (*p < 0.05; **p < 0.01). +Significant difference compared to CP from ACSF rats (^+^*p* < 0.05; ++*p* < 0.01 *p* < 0.01).

Immunostaining for TNFα showed a significantly increased intensity 3 (*p* < 0.01), 7 (*p* < 0.01) and 14 days (*p* < 0.01) following SAH induction compared to naïve animals. Increased immunofluorescence of TNFα was also found 1 (*p* < 0.05), 3 (*p* < 0.05), 7 (*p* < 0.01) and 14 days (*p* < 0.05) after ACSF application when compared to naïve animals. The intensity of TNFα immunofluorescence was significantly increased 7 days after SAH induction of when compared to ACSF animals at the same time point (< 0.05) (Figures 3A,B; Supplementary Figures 5SA–I).

IL-1β immunofluorescence intensity increased 3 (*p* < 0.01) and 7 days (*p* < 0.01) after SAH induction of as well as 3 days (*p* < 0.01) following ACSF application compared to naïve animals. Moreover, the intensity of IL-1β was significantly higher 7 (*p* < 0.01) and 14 days (*p* < 0.01) in SAH group compared with ACSF animals at the same time point. IL-1β immunofluorescence intensity was the highest in animals 7 days after SAH induction and 3 days after ACSF application (Figures 3D,E; Supplementary Figures 6SA–I).

CCL2 immunoflourescence intensity was significantly decreased 1 (*p* < 0.01), 3 (*p* < 0.01) 7 (*p* < 0.01) and 14 days (*p* < 0.01) after SAH induction as well as 1 (*p* < 0.01), 3 (p < 0.01) and 14 days (*p* < 0.01) after ACSF application compared to naïve animals. In SAH animals, increased CCL2 immunofluorescence was found 1 (*p* < 0.01) and 14 days (*p* < 0.01) and a decreased intensity 7 days (*p* < 0.01) when compared to ACSF animals at the same time points (Figures 3G,H; Supplementary Figures 7SA–I).

### 3.3 Analysis of the number of CCR2 and CXCR1 positive cells in the choroid plexus

CCR2 and CXCR1 positive cells were found mainly on the ventricular side of the cuboidal epithelial cells in the epiplexus position.

**Figure 4:**
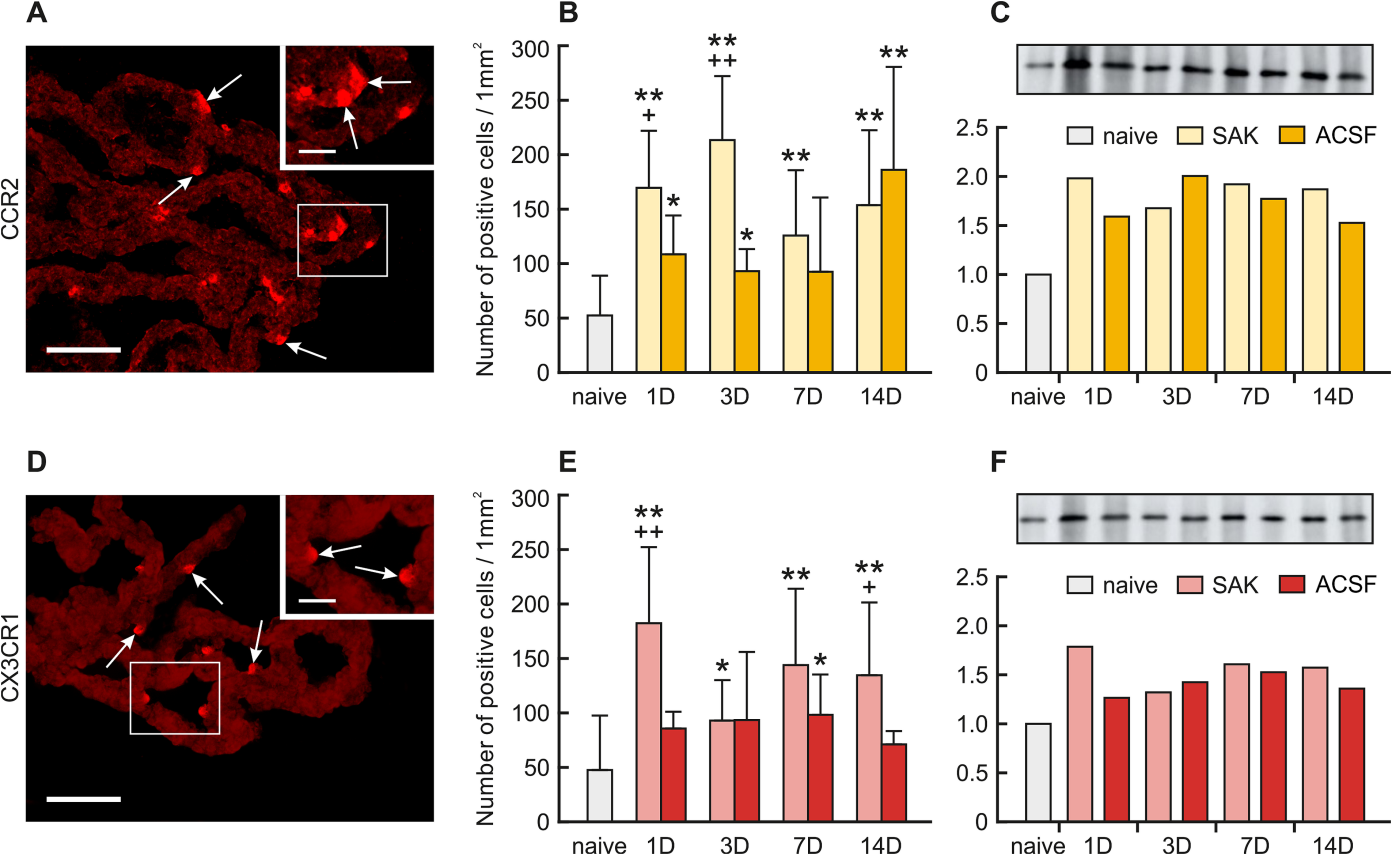
Representative merged pictures with Hoechst-stained nuclei showing CCR2 and CX3CR1 immunostaining in the CP after induction of SAH. Arrows show CCR2, **(A)** and CX3CR1 **(D)** immunopositive CP epiplexal cells. Insets at top right show higher magnifications of the regions indicated by the box in the main picture. Scale bars – main images 80 μm; insets 20 μm. Graphs show the numbers of CCR2 **(B)** and CX3CR1 **(E)** immunopositive cells (±SD) in the CP. Representative Western blot analysis comparing the levels of CCR2 (C) and CX3CR1 **(F)** in the CP from different experimental groups. *Significant difference compared to CP from naïve rats (*p < 0.05; **p < 0.01). +Significant difference compared to CP from ACSF rats (^+^*p* < 0.05; ^++^*p* < 0.01 *p* < 0.01).

The number of CCR2 positive Kolmer cells was significantly increased in the choroid plexus 1 (*n* = 169.53 ± 52.51/mm2; *p* < 0.01), 3 (*n* = 213.34 ± 58.83/mm2; *p* < 0.01), 7 (125.79 ± 59.96; *p* < 0.01) and 14 days (*n* = 153.55 ± 68.97/mm2; *p* < 0.01) following SAH induction when compared to naïve animals (*n* = 52.32 ± 36.53/mm2). A higher number of CCR2 positive cells was also found 1 (*n* = 108.53 ± 35.75; *p* < 0.05), 3 (*n* = 93.00 ± 20.34; *p* < 0.05) and 14 days (*n* = 186.13 ± 94.50; *p* < 0.01) following ACSF application compared to naïve animals (*n* = 52.32 ± 36.53/mm2). The number of CCR2 positive cells also increased 1 (*p* < 0.05) and 3 days (*p* < 0.01) after SAH when compared to ACSF groups at the same time points (Figures 4A,B; Supplementary Figures 8SA–I).

The number of CXCR1 positive cells in the CP significantly increased 1 (*n* = 182.31 ± 70.03 /mm2; *p* < 0.01), 3 (*n* = 92.92 ± 37.11/mm2; *p* < 0.05), 7 (*n* = 143.84 ± 70.12/mm2; *p* < 0.01) and 14 days (*n* = 134.58 ± 66.78; *p* < 0.01) following SAH compared to naïve animals (*n* = 47.52 ± 50.09).

Immunostaining of CXCR1 positive cells in ACSF animals displayed a significantly higher number compared to naïve animals (*n* = 47.52 ± 50.09/mm2) in the CP 7 days (*n* = 98.22 ± 37.04/mm2; *p* < 0.05) after operation with a decline thereafter. We also observed significantly increased numbers of CXCR1 positive cells in the CP 1 (*p* < 0.01) and 14 days (*p* < 0.05) after SAH compared to ACSF groups at the same time points. (Figures 4D,E; Supplementary Figure S9A–I).

### 3.4 Levels of TLR4, TLR9, FPR2, TNFα, CCL2, IL-1β, CCR2, and CXCR1 proteins in the choroid plexus following SAH induction or ACSF application

Changes in TLR4, TLR9, FPR2, TNFα, CCL2, IL-1β as well as CCR2 and CXCR1 in the choroid plexus of SAH and ACSF animals were analyzed using Western blot (Supplementary Figure S1).

The level of TLR4 protein increased 3, 7 and 14 days following SAH induction (1.4-fold; 1.3-fold and 1.3-fold) and ACSF application (1.5-fold; 1.5-fold dnd 1.4-fold) compared to naive animals (Figure 2C).

Increased TLR9 protein levels compared to naive animals were found in all periods after SAH and ACSF application the maximum seen at 7 days following SAH induction (3.1-fold) (Figure 2F). Levels of FPR2 were only slightly increased in all periods after SAH induction or ACSF application compared to naive CP (Figure 2I).

TNFα protein levels increased mainly 3 (2.0-fold), 7 (1.5-fold) and 14 days (1.7-fold) after SAH and 1 (2.0-fold) and 14 days (1.8-fold) following ACSF application when compared to naive animals (Figure 3C).

Increased levels of IL-1β protein were found 3 (1.5-fold; 1.6-fold) and 7 days (1.6-fold; 1.4-fold) following SAH or ACSF application compared to naive CP (Figure 3F).

CCL2 protein was lower compared to naive CP in all periods following SAH induction (Figure 3I). Following ACSF application we found that CCL2 levels were increased at 7 days (1.7-fold) compared to naive animals (Figure 3I).

Protein levels of CCR2 as well as CX3CR1 were also increased in all groups of SAH and ACSF animals. We found an increased number of CCR2 positive cells 1 (2.0-fold), 3 (1.7-fold), 7 (1.9-fold) and 14 days (1.9-fold) after SAH induction compared to naive animals. We also found an increased number of CCR2 positive cells 1 (1.6-fold), 3 (2.0-fold), 7 (1.8-fold) and 14 days (1.5-fold) following ACSF application compared to naive animals (Figure 4C). Increased levels of CX3CR1 protein were found mainly 1 (1.8-fold), 7 (1.6-fold) and 14 days (1.6-fold) following SAH as well as 7 days (1.5-fold) following ACSF application compared to CP from naive animals (Figure 4F).

## 4 Discussion

**Figure 5:**
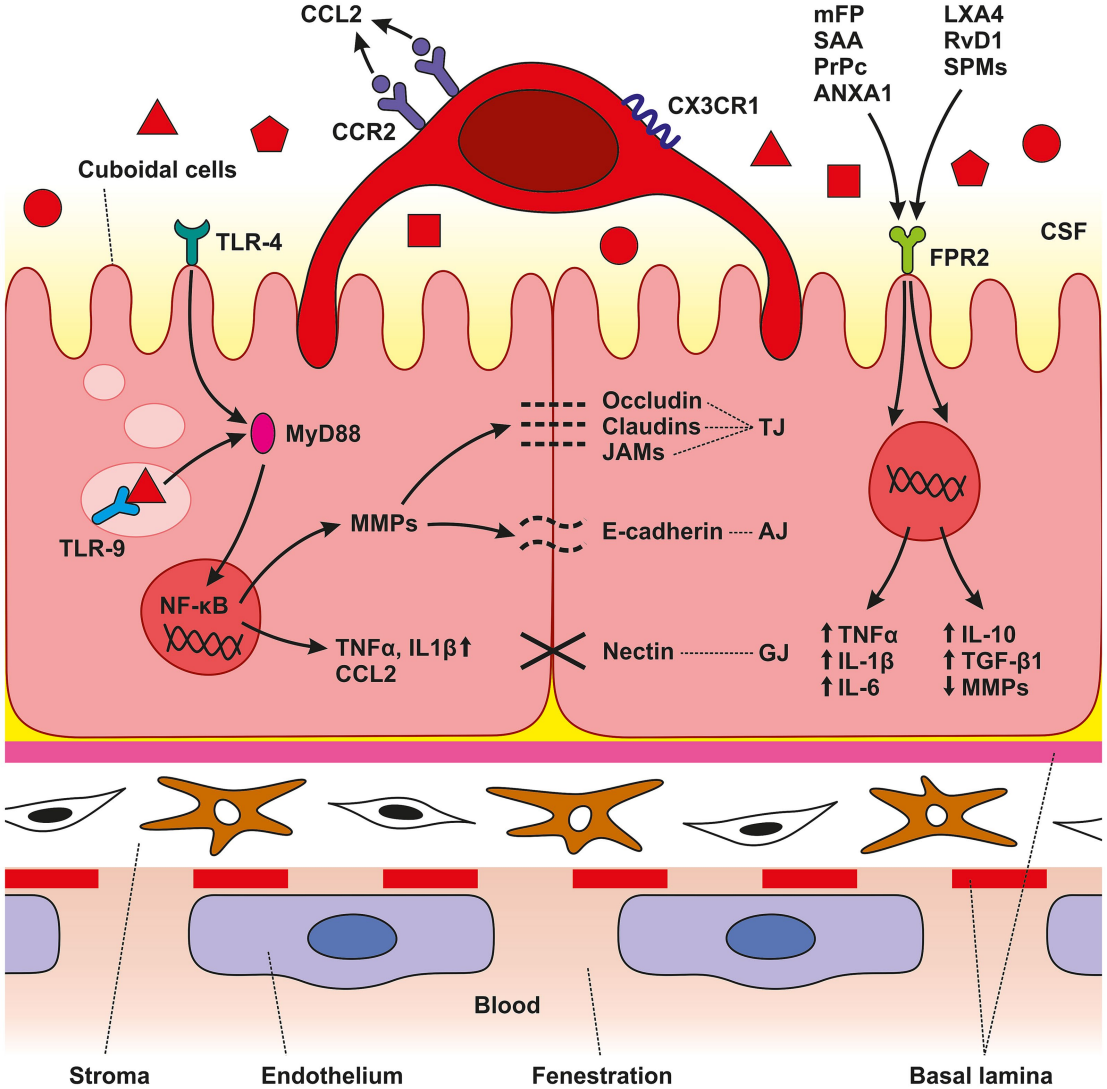
Schematic illustration of CP anatomical organization. The CP is an epithelio-endothelial convolute, comprising a highly vascularized stroma with fenestrated endothelial cells, connective tissue, and a continuous lining of epithelial cells with adhering epiplexal or Kolmer cells. We observed increased amounts of TLR4, TLR9, and FPR3 in CP epithelial cells after SAH induction. Activation of these receptors leads to the initiation of intracellular cascades resulting in accumulation of pro-inflammatory molecules including TNFα, CCL2, and IL-1β. The lower amount of the cytokine CCL2 is probably related to its consumption by binding to the CCR2 receptor, the amount of which was increased, as were CX3CR1 receptors. SAH induction resulted in increased CCR2 and CX3CR1 immunopositivity only on the surface of epiplexal Kolmer cells.

In the study presented we show elevated levels of the DAMP receptors, TLR4, TLR9 and FPR2 in the CP epithelial cells at various time points following SAH induction or ACSF application of. The increased levels of pro-inflammatory receptors were associated with upregulation of TNFα and IL-1β, both powerful proinflammatory cytokines, as well as the accumulation of macrophages expressing CCR2 and CXCR1 in the CP epiplexus position (Figure 5). Intraventricular hemorrhage or SAH triggers a pro-inflammatory reaction resulting in the disruption of the blood–brain barrier (Solár et al., 2020c, 2022). Inflammatory pathways are initiated in the first few seconds following aneurysm rupture and they continue to remain activated even in later stages. Cerebrovascular inflammation both in the early and the later course of SAH contributes to the development of the principal complications such as vasospasms and hydrocephalus (Provencio, 2013; Solar et al., 2022; Solár et al., 2022). Blood elements and blood-degradation products in the CSF are in direct contact with epithelial and epiplexus cells/KC, of the CP (Liszczak et al., 1984). SAH induced inflammation involves adhesion molecules, cytokines, migration of immune cells and complement activation (Sehba et al., 2011; Miller et al., 2014). These changes in the CP are linked to the abnormal functioning of the B-CSF- barrier (Simard et al., 2011; Xiang et al., 2017). Although several pathways have been proposed, the exact source of these pro-inflammatory molecules and the increased number of immune cells in the CP following SAH is not known. Circulating monocytes could migrate into the CP via fenestrated capillaries and pass through the epithelial cells by “emperipolesis” to the ventricular side of the epithelial cells and become KC (Ling et al., 1998). Proliferation of ED2+ macrophages may be another source of increased macrophage number after SAH (Solár et al., 2020b). Moreover, meningeal vessels may also contribute to the accumulation of immune cells in the CP following SAH (Wilson et al., 2010). Pathophysiological cascades induced by SAH are linked to blood degradation products in the CSF which serve as alarmins called DAMPs (Miyake, 2007; Pan et al., 2020). DAMPs can activate TLRs which play an important role in innate immunity and the development of neuroinflammation (Lin et al., 2010; Kwon et al., 2015). TLR4, a transmembrane protein, and TLR9, located in intracellular vesicles of the endoplasmic reticulum, lysosomes, and endosomes were found to be expressed primarily in microglia, but also on other cells including astrocytes, neurons, endothelial cells as well as CP epithelial cells (Buchanan et al., 2010; Skipor et al., 2015; Benbenishty et al., 2019). The prototypical ligand for TLR4 is bacterial lipopolysaccharide (LPS) (Vallières et al., 2006). Similarly, mitochondrial DNA which is similar to bacterial DNA has unmethylated CpG DNA motifs which act as ligands for TLR9 (Zhang et al., 2010). In addition to their typical ligands, TLRs are known for their capacity to recognize many other DAMPs or PAMPs and activate the immune system leading to inflammation (Paradis et al., 2016; Li et al., 2020; Metzemaekers et al., 2020). Activation of TLRs by DAMPs following hemorrhagic stroke has been reported. An experimental study using the intracerebral hemorrhage model showed that activation of TLR-9 increased Iba-1 and HO-1 positive cells with enlarged cell bodies around the hematoma thus facilitating macrophage/microglial phagocytosis (Wei et al., 2022). Studies focused on TLR4 revealed elevated activation of two inflammatory cell-signaling pathways, MAPK and NF-κB pathways (Khey et al., 2020). Activation of MAPK leads to the phosphorylation of transcription factors resulting in proinflammatory effects including production of proinflammatory molecules and chemotaxis. Intracellular MAPK-activated signaling pathways involve the expression of cytokines and chemokines such as IL-1β, IL-6, IL-8, TNF-*α*, and MCP-1 (Sasaki et al., 2004). This is in accordance with our finding of the increased amount of IL-1β and TNF-α mainly 3- and 7-days following induction of SAH as well as application of ACSF with a decrease to near-normal values after 14 days. Activation of NF-κB was also increased after induction of SAH in experimental animals (Khey et al., 2020). Following SAH and subsequent activation of TLRS by various DAMPs, NF-κB translocates into the nucleus and upregulates the expression of pro-inflammatory molecules, cell adhesion molecules, TNF-α, COX, and others (Okada and Suzuki, 2017). Moreover, studies focused on TLR4 revealed that DAMPs lead to the activation of myeloid differentiation primary response protein 88 (MyD88)-dependent NF-κB signaling cascade resulting in increased secretion of TNFα (Figueiredo et al., 2007, p. 4; Karimy et al., 2020). TNFα induces DNA fragmentation, chromatin condensation and activation of caspase-3 resulting in apoptosis in the CP. These changes decrease the transepithelial membrane potential and increase paracellular permeability of the B-CSF barrier. In parallel, the cytotoxic effect of TNFα characterized by LDH-release and the detachment of HMGB1 from chromatin in CP epithelial cells indicates post-apoptotic events or secondary necrosis. Therefore, it seems that not only apoptosis but also necrosis plays a role in the TNFα-induced alteration of the B-CSF-barrier (Silva et al., 2008; Schwerk et al., 2010). In addition, up-regulation of TNFα induces CSF secretion mediated by the STE20/SPS1-related proline/alanine-rich kinase (SPAK) – Sodium Potassium Chloride cotransporter-1 (NKCC1) complex on the apical membrane of the CP epithelial cells. TNFα as well as interferon *γ* (IFN-γ) stimulate SPAK signaling through the NF-κB-dependent pathway, leading to increased ion transport in epithelial cells resulting in CSF hypersecretion (Karimy et al., 2017; Gregoriades et al., 2019). Moreover, activation of TLR4/NF-κB/TNFα signaling cascade upregulates the expression of other strong chemotactic and pro-inflammatory effector molecules including CCL2 and IL-1β (Gram et al., 2014). These proinflammatory molecules are critical for monocyte trafficking into the CP leading to hypersecretion of CSF (Solár et al., 2020c). The CP is a unique structure through which immune cells can pass into the CNS as a response to various inflammatory or non-inflammatory pathologies. The role of chemokines and cytokines in the trafficking of immune cells through the CP is currently attracting considerable research interest. CCL2/CCR2 signaling plays an important role in the migration of peripheral immune cells in the brain during neuro-inflammation but also in the systemic inflammatory reaction (Cazareth et al., 2014; Llovera et al., 2017; Shimada and Hasegawa-Ishii, 2021). The presence of blood and blood degradation products in the CSF is accompanied by increased expression of pro-inflammatory and chemotactic effector molecules including CCL2 and TNFα (Gram et al., 2014). Following the activation of the CCL2/CCR2 signaling cascade, both proinflammatory and anti-inflammatory actions have been observed—and the response depends on the disease (Deshmane et al., 2009). CCL2 ligand expression was found in neurons, astrocytes, perivascular microglia and infiltrating leukocytes. An important role for CCL2 has been confirmed in various diseases including multiple sclerosis, stroke, Alzheimer’s disease, acute brain injury and experimental autoimmune encephalitis (Mildner et al., 2009; Conductier et al., 2010; Saederup et al., 2010). The CP responds to different stimuli by increased CCL2 mediating the transmigration of CCR2+ leukocytes (Thibeault et al., 2001; Reyes et al., 2003). Secretion of CCL2 in the CP epithelium occurs throughout the apical as well as the basolateral membranes and acts as a strong chemoattractant (Mahad et al., 2006; Kaur et al., 2016). However, in our study, the level of CCL2 was slightly decreased following SAH or ACSF application. A possible explanation for this is that CCL2 is consumed by the increasing number of CCR2+ cells (Mahad et al., 2006). Moreover, there is some evidence that constitutively internalized and recycled CCR2 removes CCL2 from the cellular environment (Zhao et al., 2019). Based on this finding, it can be assumed that the increased number of CCR2+ macrophages removeCCL2 from the CP microenvironment. Another possible explanation is that CCL2 is not necessary for macrophage accumulation. Other mechanisms, or other chemokines may be involved, such as resident macrophage proliferation and CCR2-independent monocyte recruitment (Talsma et al., 2022).

CCR2 and CX3CR1 are membrane receptors involved in trafficking along inflamed vessels resulting in invasion and accumulation of circulating monocytes in the CNS. Therefore, accumulation of CCR2 and CX3CR1 positive macrophages in the CP indicates their origin from peripheral monocytes (Combadiere et al., 1998; Auffray et al., 2007; Prinz and Priller, 2010; Meghraoui-Kheddar et al., 2020). Monocytes expressing CCR2 and CX3CR1, the so-called “inflammatory” monocytes, are highly mobile and larger in size compared to “resident” monocytes (Prinz and Priller, 2010). Several hypotheses have been put forward to explain how circulating monocytes can become CP epiplexus cells. The most-widely accepted one proposes that circulating monocytes can enter the CP through the fenestrated capillaries and pass through the epithelial cells by “emperipolesis” to the ventricular side of the epithelial cells to become CP epiplexus cells (Ling et al., 1998). Moreover, meningeal vessels may also contribute to the accumulation of macrophages in the epiplexus position following SAH (Wilson et al., 2010). FPR2, an innate immune receptor, may play a role in the orchestration and polarization of CP macrophages following SAH. Immunohistochemical studies showed the expression of FPRs in a different part of the CNS including CP epithelial cells (Becker et al., 1998). Mitochondrial N-formylated peptides released from injured and disintegrated cells following tissue damage act as key ligands for FPRs (Wei et al., 2020). Activation of FPR2 leads to dual effects, pro-inflammatory or anti-inflammatory activity based on the types of endogenous ligands. Activation of FPR2 by different endogenous ligands such as Serum Amyloid A (SAA), Prion Protein (PrPc), Annexin A1 (ANXA1) or mitochondrial formyl peptides (mFP) derived from damaged mitochondria, exert pro-inflammatory reactions characterized by the elevated expression of pro-inflammatory molecules like TNFα, IL-1β and IL-6. On the other hand, ligands such as Lipoxin A4 (LXA4), Resolvin D1 (RvD1) or Specialized Pro-resolving Mediators (SPMs), induce the inhibition of pro-inflammatory cytokine production, suppression of metalloproteinases, upregulation of anti-inflammatory cytokines including IL-10 and transforming growth factor-β1 (TGF- β1), as well as enhancing the clearing capacity of macrophages (Dufton and Perretti, 2010; Guo et al., 2016; Wenceslau et al., 2016; Tylek et al., 2021). Following SAH, some cellular components such as mFP released from damaged mitochondria act as DAMPs and contribute to the development of neuroinflammation (Zhang et al., 2022). FPR2 mediates a pro-inflammatory response upon binding to mFP. In experimantal models of various diseases, FPR2 mediates extracellular signals into the cell through the ERK1/2 pathway. ERK1/2 activates downstream transcription factors including c-myc, c-jun, and NF-κB leading to the expression of inflammatory cytokines (Cao et al., 2023). On the other hand, the protective and anti-inflammatory role of FPR2 is probably associated with M2 macrophages, which potentiate tissue repair and regeneration (Yuan et al., 2022). In our previous work, we found a higher number of M2 macrophages in the CP following SAH induction mainly in the later stages (Solár et al., 2020b). The ability of CP epithelial cells to increase the FPR2 number may be one of the mechanisms leading to the accumulation of macrophages with an anti-inflammatory M2 phenotype. This may contribute to restoring CNS homeostasis after SAH. However, in the current study, we find that the expression of FPR2 on the surface of CP epithelial cells was only slightly increased. Thus, the role of FPR2 in the development of either a pro-inflammatory or anti-inflammatory reaction in the CP following SAH is probably marginal. As for increased ICP following SAH, we found increased immunofluorescence intensities for TLR4, TLR9 and TNFα in the CP after ACSF application. It is known that ICP rises immediately after aneurysm rupture (Prunell et al., 2002). Here, we mimic increased ICP following SAH by applying ACSF into the cisterna magna. Intrathecal application of ACSF is widely used as a control in experimental models without evidence of immunogenicity (Hylden and Wilcox, 1980; Hernangómez et al., 2016). Despite these findings, we showed that increased ICP induced by ACSF application leads to immune cell accumulation in the CP. The exact mechanism of how elevated ICP contributes to cellular and molecular inflammatory changes in the CP is not known. However, based on our results it can be assumed that TLR4 and TLR9 respond not only to blood degradation products but also to increased ICP following aneurysm rupture and extravasation of blood into the subarachnoid space. Activation of Piezo channels—mechanosensitive ion channels located in the cell membrane – might be potentially involved. Activation of Piezo channels by mechanical stretching leads to the influx of extracellular Ca2+ (Liu et al., 2022). In addition, it was revealed that these channels cooperate with TLR4 to promote their response (Geng et al., 2021). Moreover, Piezo mechanoreceptors are also involved in immune cell response regulation (Liu et al., 2018).

Our study has several limitations. First, we used an experimental model of SAH with the application of blood into the cisterna magna that mimics the bleeding into the subarachnoid space in humans. Due to the deep location of the CP in the brain, using an experimental model of SAH is the only possibility to describe the changes in the B-CSF barrier. However, using an animal experimental model partially limits the application of pathophysiological changes to humans. Furthermore, we must consider the absence of aneurysm rupture, including the fact that the cellular and subcellular pathways may differ in mammals and rodents. An alternative is to use *in vitro* models; however, these models lack the interaction of surrounding tissues, including blood and CSF. Another limitation is the use of only adult male rats. The main reason for using male rats rather than female ones is that fluctuating female sex hormones may generate less controlled experimental conditions and influence the result. Therefore, to have the experiment as standardized as possible, based on current studies, we used male rats. In our study, to mimic elevated ICP following SAH, we used artificial CSF according to the composition that is widely used in studies. Prunell et al. (2002). referred that increased ICP following the application of ACSF mimics the SAH. However, the absence of other cellular and molecular components from blood may limit the changes caused by the increased ICP following SAH.

In the current study, we observed changes in the amount of different TLRs and proinflammatory molecules also in the animals after ACSF application, but these changes were less pronounced compared to SAH animals. Therefore, it can be assumed that the changes after the ACSF application were mainly caused by elevated ICP.

Our results outline a possible mechanism for the accumulation of immune cells in the CP following SAH. TLRs, mainly TLR9, probably play an important role in the development of inflammatory changes in CP epithelial cells. Activation of TLRs leads to the elevation of chemotactic molecules in the CP and subsequent trafficking of peripheral monocytes. Importantly, increased ICP induced by ACSF application also leads to increased expression of pro-inflammatory cytokines as well as an increased number of monocyte-derived macrophages. These findings provide the basis for further studies to develop potential new therapeutic tools. In particular, the reduction of inflammatory changes may attenuate the development of post-hemorrhagic hydrocephalus and other complications after SAH. Our results suggest that not only blood components but also acute increase in ICP contribute to the development of the complex inflammatory reaction in the CP that eventually leads to the alteration in the B-CSF barrier.

## Data Availability

The original contributions presented in the study are included in the article/Supplementary material, further inquiries can be directed to the corresponding author.

## References

[ref1] Akira S. Uematsu S. Takeuchi O. (2006). Pathogen recognition and innate immunity. Cell 124, 783–801. doi: 10.1016/j.cell.2006.02.015 16497588

[ref2] Auffray C. Fogg D. Garfa M. Elain G. Join-Lambert O. Kayal S.. (2007). Monitoring of blood vessels and tissues by a population of monocytes with patrolling behavior. Science 317, 666–670. doi: 10.1126/science.1142883, PMID: 17673663

[ref3] Becker E. L. Forouhar F. A. Grunnet M. L. Boulay F. Tardif M. Bormann B. J.. (1998). Broad immunocytochemical localization of the formylpeptide receptor in human organs, tissues, and cells. Cell Tissue Res. 292, 129–135. doi: 10.1007/s004410051042, PMID: 9506920

[ref4] Bederson J. B. Levy A. L. Ding W. H. Kahn R. DiPerna C. A. Jenkins A. L.. (1998). Acute vasoconstriction after subarachnoid hemorrhage. Neurosurgery 42, 352–362. doi: 10.1097/00006123-199802000-00091, PMID: 9482187

[ref5] Benbenishty A. Gadrich M. Cottarelli A. Lubart A. Kain D. Amer M.. (2019). Prophylactic TLR9 stimulation reduces brain metastasis through microglia activation. PLoS Biol. 17:e2006859. doi: 10.1371/journal.pbio.2006859, PMID: 30921319 PMC6469801

[ref6] Brazda V. Muller P. Brozkova K. Vojtesek B. (2006). Restoring wild-type conformation and DNA-binding activity of mutant p53 is insufficient for restoration of transcriptional activity. Biochem. Biophys. Res. Commun. 351, 499–506. doi: 10.1016/j.bbrc.2006.10.065, PMID: 17070499

[ref7] Buchanan M. M. Hutchinson M. Watkins L. R. Yin H. (2010). Toll-like receptor 4 in CNS pathologies. J. Neurochem. 114, 13–27. doi: 10.1111/j.1471-4159.2010.06736.x, PMID: 20402965 PMC2909662

[ref8] Cao Y. Chen J. Liu F. Qi G. Zhao Y. Xu S.. (2023). Formyl peptide receptor 2 activation by mitochondrial formyl peptides stimulates the neutrophil proinflammatory response via the ERK pathway and exacerbates ischemia-reperfusion injury. Cell. Mol. Biol. Lett. 28:4. doi: 10.1186/s11658-023-00416-1, PMID: 36658472 PMC9854225

[ref9] Cazareth J. Guyon A. Heurteaux C. Chabry J. Petit-Paitel A. (2014). Molecular and cellular neuroinflammatory status of mouse brain after systemic lipopolysaccharide challenge: importance of CCR2/CCL2 signaling. J. Neuroinflammation 11:132. doi: 10.1186/1742-2094-11-132, PMID: 25065370 PMC4237883

[ref10] Combadiere C. Salzwedel K. Smith E. D. Tiffany H. L. Berger E. A. Murphy P. M. (1998). Identification of CX3CR1. A chemotactic receptor for the human CX3C chemokine fractalkine and a fusion coreceptor for HIV-1. J. Biol. Chem. 273, 23799–23804. doi: 10.1074/jbc.273.37.23799, PMID: 9726990

[ref11] Conductier G. Blondeau N. Guyon A. Nahon J.-L. Rovère C. (2010). The role of monocyte chemoattractant protein MCP1/CCL2 in neuroinflammatory diseases. J. Neuroimmunol. 224, 93–100. doi: 10.1016/j.jneuroim.2010.05.010, PMID: 20681057

[ref12] Connolly E. S. Rabinstein A. A. Carhuapoma J. R. Derdeyn C. P. Dion J. Higashida R. T.. (2012). Guidelines for the management of aneurysmal subarachnoid hemorrhage: a guideline for healthcare professionals from the American Heart Association/american Stroke Association. Stroke 43, 1711–1737. doi: 10.1161/STR.0b013e3182587839 22556195

[ref13] d’Avella D. Cicciarello R. Zuccarello M. Albiero F. Romano A. Angileri F. F.. (1996). Brain energy metabolism in the acute stage of experimental subarachnoid haemorrhage: local changes in cerebral glucose utilization. Acta Neurochir. 138:737–743; discussion 744. doi: 10.1007/BF01411481, PMID: 8836291

[ref14] Deshmane S. L. Kremlev S. Amini S. Sawaya B. E. (2009). Monocyte chemoattractant Protein-1 (MCP-1): an overview. J. Interf. Cytokine Res. 29, 313–326. doi: 10.1089/jir.2008.0027, PMID: 19441883 PMC2755091

[ref15] Dubový P. Brázda V. Klusáková I. Hradilová-Svíženská I. (2013). Bilateral elevation of interleukin-6 protein and mRNA in both lumbar and cervical dorsal root ganglia following unilateral chronic compression injury of the sciatic nerve. J. Neuroinflammation 10:55. doi: 10.1186/1742-2094-10-55, PMID: 23634725 PMC3657546

[ref16] Dubový P. Klusáková I. Svízenská I. (2002). A quantitative immunohistochemical study of the endoneurium in the rat dorsal and ventral spinal roots. Histochem. Cell Biol. 117, 473–480. doi: 10.1007/s00418-002-0411-5, PMID: 12107498

[ref17] Dufton N. Perretti M. (2010). Therapeutic anti-inflammatory potential of formyl-peptide receptor agonists. Pharmacol. Ther. 127, 175–188. doi: 10.1016/j.pharmthera.2010.04.010, PMID: 20546777

[ref18] Etminan N. Chang H.-S. Hackenberg K. de Rooij N. K. Vergouwen M. D. I. Rinkel G. J. E.. (2019). Worldwide incidence of aneurysmal subarachnoid hemorrhage according to region, time period, blood pressure, and smoking prevalence in the population: A systematic review and Meta-analysis. JAMA Neurol. 76, 588–597. doi: 10.1001/jamaneurol.2019.0006, PMID: 30659573 PMC6515606

[ref19] Figueiredo R. T. Fernandez P. L. Mourao-Sa D. S. Porto B. N. Dutra F. F. Alves L. S.. (2007). Characterization of heme as activator of toll-like receptor 4. J. Biol. Chem. 282, 20221–20229. doi: 10.1074/jbc.M610737200, PMID: 17502383

[ref20] Geng J. Shi Y. Zhang J. Yang B. Wang P. Yuan W.. (2021). TLR4 signalling via Piezo1 engages and enhances the macrophage mediated host response during bacterial infection. Nat. Commun. 12:3519. doi: 10.1038/s41467-021-23683-y, PMID: 34112781 PMC8192512

[ref21] Gram M. Sveinsdottir S. Cinthio M. Sveinsdottir K. Hansson S. R. Mörgelin M.. (2014). Extracellular hemoglobin - mediator of inflammation and cell death in the choroid plexus following preterm intraventricular hemorrhage. J. Neuroinflammation 11:200. doi: 10.1186/s12974-014-0200-9, PMID: 25441622 PMC4269927

[ref22] Gregoriades J. M. C. Madaris A. Alvarez F. J. Alvarez-Leefmans F. J. (2019). Genetic and pharmacological inactivation of apical Na+-K+-2Cl- cotransporter 1 in choroid plexus epithelial cells reveals the physiological function of the cotransporter. Am. J. Physiol. Cell Physiol. 316, C525–C544. doi: 10.1152/ajpcell.00026.2018, PMID: 30576237 PMC6482671

[ref23] Guo Z. Hu Q. Xu L. Guo Z.-N. Ou Y. He Y.. (2016). Lipoxin A4 reduces inflammation through formyl peptide receptor 2/p38 MAPK signaling pathway in subarachnoid hemorrhage rats. Stroke 47, 490–497. doi: 10.1161/STROKEAHA.115.011223, PMID: 26732571 PMC4729632

[ref24] Hernangómez M. Klusáková I. Joukal M. Hradilová-Svíženská I. Guaza C. Dubový P. (2016). CD200R1 agonist attenuates glial activation, inflammatory reactions, and hypersensitivity immediately after its intrathecal application in a rat neuropathic pain model. J. Neuroinflammation 13, 43–15. doi: 10.1186/s12974-016-0508-8, PMID: 26891688 PMC4759712

[ref25] Hylden J. L. Wilcox G. L. (1980). Intrathecal morphine in mice: a new technique. Eur. J. Pharmacol. 67, 313–316. doi: 10.1016/0014-2999(80)90515-4 6893963

[ref26] Iwasaki A. Medzhitov R. (2004). Toll-like receptor control of the adaptive immune responses. Nat. Immunol. 5, 987–995. doi: 10.1038/ni1112 15454922

[ref27] Jackowski A. Crockard A. Burnstock G. Russell R. R. Kristek F. (1990). The time course of intracranial pathophysiological changes following experimental subarachnoid haemorrhage in the rat. J. Cereb. Blood Flow Metab. 10, 835–849. doi: 10.1038/jcbfm.1990.140, PMID: 2211877

[ref28] Karimy J. K. Reeves B. C. Kahle K. T. (2020). Targeting TLR4-dependent inflammation in post-hemorrhagic brain injury. Expert Opin. Ther. Targets 24, 525–533. doi: 10.1080/14728222.2020.1752182, PMID: 32249624 PMC8104018

[ref29] Karimy J. K. Zhang J. Kurland D. B. Theriault B. C. Duran D. Stokum J. A.. (2017). Inflammation-dependent cerebrospinal fluid hypersecretion by the choroid plexus epithelium in posthemorrhagic hydrocephalus. Nat. Med. 23, 997–1003. doi: 10.1038/nm.4361, PMID: 28692063

[ref30] Kaur C. Rathnasamy G. Ling E.-A. (2016). The choroid plexus in healthy and diseased brain. J. Neuropathol. Exp. Neurol. 75, 198–213. doi: 10.1093/jnen/nlv030, PMID: 26888305

[ref31] Khey K. M. W. Huard A. Mahmoud S. H. (2020). Inflammatory pathways following subarachnoid hemorrhage. Cell. Mol. Neurobiol. 40, 675–693. doi: 10.1007/s10571-019-00767-4 31808009 PMC11448815

[ref32] Kwon M. S. Woo S. K. Kurland D. B. Yoon S. H. Palmer A. F. Banerjee U.. (2015). Methemoglobin is an endogenous toll-like receptor 4 ligand-relevance to subarachnoid hemorrhage. Int. J. Mol. Sci. 16, 5028–5046. doi: 10.3390/ijms16035028 25751721 PMC4394463

[ref33] Li L. Ni L. Heary R. F. Elkabes S. (2020). Astroglial TLR9 antagonism promotes chemotaxis and alternative activation of macrophages via modulation of astrocyte-derived signals: implications for spinal cord injury. J. Neuroinflammation 17, 1–18. doi: 10.1186/s12974-020-01748-x, PMID: 32098620 PMC7041103

[ref34] Lin T. Kwak Y. H. Sammy F. He P. Thundivalappil S. Sun G.. (2010). Synergistic inflammation is induced by blood degradation products with microbial toll-like receptor agonists and is blocked by Hemopexin. J. Infect. Dis. 202, 624–632. doi: 10.1086/654929, PMID: 20617898 PMC2932749

[ref35] Ling E. A. Kaur C. Lu J. (1998). Origin, nature, and some functional considerations of intraventricular macrophages, with special reference to the epiplexus cells. Microsc. Res. Tech. 41, 43–56. doi: 10.1002/(SICI)1097-0029(19980401)41:1<43::AID-JEMT5>3.0.CO;2-V, PMID: 9550136

[ref36] Liszczak T. M. Black P. M. Tzouras A. Foley L. Zervas N. T. (1984). Morphological changes of the basilar artery, ventricles, and choroid plexus after experimental SAH. J. Neurosurg. 61, 486–493. doi: 10.3171/jns.1984.61.3.0486, PMID: 6747684

[ref37] Liu H. Hu J. Zheng Q. Feng X. Zhan F. Wang X.. (2022). Piezo1 channels as force sensors in mechanical force-related chronic inflammation. Front. Immunol. 13:816149. doi: 10.3389/fimmu.2022.816149, PMID: 35154133 PMC8826255

[ref38] Liu C. S. C. Raychaudhuri D. Paul B. Chakrabarty Y. Ghosh A. R. Rahaman O.. (2018). Cutting edge: Piezo1 Mechanosensors optimize human T cell activation. J. Immunol. 200, 1255–1260. doi: 10.4049/jimmunol.1701118, PMID: 29330322

[ref39] Llovera G. Benakis C. Enzmann G. Cai R. Arzberger T. Ghasemigharagoz A.. (2017). The choroid plexus is a key cerebral invasion route for T cells after stroke. Acta Neuropathol. 134, 851–868. doi: 10.1007/s00401-017-1758-y, PMID: 28762187

[ref40] Mahad D. Callahan M. K. Williams K. A. Ubogu E. E. Kivisäkk P. Tucky B.. (2006). Modulating CCR2 and CCL2 at the blood-brain barrier: relevance for multiple sclerosis pathogenesis. Brain 129, 212–223. doi: 10.1093/brain/awh655, PMID: 16230319

[ref41] Matz P. Turner C. Weinstein P. R. Massa S. M. Panter S. S. Sharp F. R. (1996). Heme-oxygenase-1 induction in glia throughout rat brain following experimental subarachnoid hemorrhage. Brain Res. 713, 211–222. doi: 10.1016/0006-8993(95)01511-6, PMID: 8724993

[ref42] Meghraoui-Kheddar A. Barthelemy S. Boissonnas A. Combadière C. (2020). Revising CX3CR1 expression on murine classical and non-classical monocytes. Front. Immunol. 11:1117. doi: 10.3389/fimmu.2020.01117, PMID: 32582197 PMC7283740

[ref43] Metzemaekers M. Gouwy M. Proost P. (2020). Neutrophil chemoattractant receptors in health and disease: double-edged swords. Cell. Mol. Immunol. 17, 433–450. doi: 10.1038/s41423-020-0412-0, PMID: 32238918 PMC7192912

[ref44] Mildner A. Mack M. Schmidt H. Brück W. Djukic M. Zabel M. D.. (2009). CCR2+Ly-6Chi monocytes are crucial for the effector phase of autoimmunity in the central nervous system. Brain 132, 2487–2500. doi: 10.1093/brain/awp144, PMID: 19531531

[ref45] Miller B. A. Turan N. Chau M. Pradilla G. (2014). Inflammation, vasospasm, and brain injury after subarachnoid hemorrhage. Biomed. Res. Int. 2014:384342, 1–16. doi: 10.1155/2014/384342, PMID: 25105123 PMC4106062

[ref46] Miyake K. (2007). Innate immune sensing of pathogens and danger signals by cell surface toll-like receptors. Semin. Immunol. 19, 3–10. doi: 10.1016/j.smim.2006.12.002, PMID: 17275324

[ref47] Okada T. Suzuki H. (2017). Toll-like receptor 4 as a possible therapeutic target for delayed brain injuries after aneurysmal subarachnoid hemorrhage. Neural Regen. Res. 12, 193–196. doi: 10.4103/1673-5374.200795, PMID: 28400792 PMC5361494

[ref48] Pan P. Xu L. Zhang H. Liu Y. Lu X. Chen G.. (2020). A review of hematoma components clearance mechanism after subarachnoid hemorrhage. Front. Neurosci. 14:685. doi: 10.3389/fnins.2020.00685, PMID: 32733194 PMC7358443

[ref49] Paradis A. Bernier S. Dumais N. (2016). TLR4 induces CCR7-dependent monocytes transmigration through the blood–brain barrier. J. Neuroimmunol. 295-296, 12–17. doi: 10.1016/j.jneuroim.2016.03.019, PMID: 27235343

[ref50] Piccinini A. M. Midwood K. S. (2010). DAMPening inflammation by modulating TLR Signalling. Mediat. Inflamm. 2010:e672395, 1–21. doi: 10.1155/2010/672395, PMID: 20706656 PMC2913853

[ref51] Prinz M. Priller J. (2010). Tickets to the brain: role of CCR2 and CX3CR1 in myeloid cell entry in the CNS. J. Neuroimmunol. 224, 80–84. doi: 10.1016/j.jneuroim.2010.05.015 20554025

[ref52] Provencio J. J. (2013). Inflammation in subarachnoid hemorrhage and delayed deterioration associated with vasospasm: A review. Acta Neurochir. Suppl. 115, 233–238. doi: 10.1007/978-3-7091-1192-5_42, PMID: 22890674 PMC3597075

[ref53] Prunell G. F. Mathiesen T. Diemer N. H. Svendgaard N.-A. (2003). Experimental subarachnoid hemorrhage: subarachnoid blood volume, mortality rate, neuronal death, cerebral blood flow, and perfusion pressure in three different rat models. Neurosurgery 52:176. doi: 10.1097/00006123-200301000-00022, PMID: 12493115

[ref54] Prunell G. F. Mathiesen T. Svendgaard N.-A. (2002). A new experimental model in rats for study of the pathophysiology of subarachnoid hemorrhage. Neuroreport 13, 2553–2556. doi: 10.1097/00001756-200212200-00034, PMID: 12499866

[ref55] Reyes T. M. Walker J. R. DeCino C. Hogenesch J. B. Sawchenko P. E. (2003). Categorically distinct acute stressors elicit dissimilar transcriptional profiles in the paraventricular nucleus of the hypothalamus. J. Neurosci. 23, 5607–5616. doi: 10.1523/JNEUROSCI.23-13-05607.2003, PMID: 12843263 PMC6741278

[ref56] Saederup N. Cardona A. E. Croft K. Mizutani M. Cotleur A. C. Tsou C.-L.. (2010). Selective chemokine receptor usage by central nervous system myeloid cells in CCR2-red fluorescent protein knock-in mice. PLoS One 5:e13693. doi: 10.1371/journal.pone.0013693, PMID: 21060874 PMC2965160

[ref57] Sasaki T. Kasuya H. Onda H. Sasahara A. Goto S. Hori T.. (2004). Role of p38 mitogen-activated protein kinase on cerebral vasospasm after subarachnoid hemorrhage. Stroke 35, 1466–1470. doi: 10.1161/01.STR.0000127425.47266.20, PMID: 15118180

[ref58] Schwerk C. Rybarczyk K. Essmann F. Seibt A. Mölleken M.-L. Zeni P.. (2010). TNFα induces choroid plexus epithelial cell barrier alterations by apoptotic and nonapoptotic mechanisms. J. Biomed. Biotechnol. 2010:307231. doi: 10.1155/2010/307231, PMID: 20369072 PMC2847764

[ref59] Sehba F. A. Pluta R. M. Zhang J. H. (2011). Metamorphosis of subarachnoid hemorrhage research: from delayed vasospasm to early brain injury. Mol. Neurobiol. 43, 27–40. doi: 10.1007/s12035-010-8155-z, PMID: 21161614 PMC3023855

[ref60] Shimada A. Hasegawa-Ishii S. (2021). Increased cytokine expression in the choroid plexus stroma and epithelium in response to endotoxin-induced systemic inflammation in mice. Toxicol. Rep. 8, 520–528. doi: 10.1016/j.toxrep.2021.03.002, PMID: 33747797 PMC7973137

[ref61] Silva M. T. do Vale A. dos Santos N. M. N. (2008). Secondary necrosis in multicellular animals: an outcome of apoptosis with pathogenic implications. Apoptosis 13, 463–482. doi: 10.1007/s10495-008-0187-8, PMID: 18322800 PMC7102248

[ref62] Simard P. F. Tosun C. Melnichenko L. Ivanova S. Gerzanich V. Simard J. M. (2011). Inflammation of the choroid plexus and ependymal layer of the ventricle following intraventricular hemorrhage. Transl. Stroke Res. 2, 227–231. doi: 10.1007/s12975-011-0070-8, PMID: 21731590 PMC3128335

[ref63] Skipor J. Szczepkowska A. Kowalewska M. Herman A. P. Lisiewski P. (2015). Profile of toll-like receptor mRNA expression in the choroid plexus in adult ewes. Acta Vet. Hung. 63, 69–78. doi: 10.1556/AVet.2014.027, PMID: 25374259

[ref64] Solár P. Brázda V. Levin S. Zamani A. Jančálek R. Dubový P.. (2020a). Subarachnoid hemorrhage increases level of Heme Oxygenase-1 and Biliverdin reductase in the choroid plexus. Front. Cell. Neurosci. 14:593305. doi: 10.3389/fncel.2020.593305, PMID: 33328892 PMC7732689

[ref65] Solar P. Joukal M. Silar C. Jancalek R. (2022). Impact of analgesic regimen on patient outcome following subarachnoid hemorrhage: positive adjuvant effects of metamizole. Br. J. Neurosurg. 38, 1304–1311. doi: 10.1080/02688697.2022.2151563, PMID: 36469604

[ref66] Solár P. Klusáková I. Jančálek R. Dubový P. Joukal M. (2020b). Subarachnoid hemorrhage induces dynamic immune cell reactions in the choroid plexus. Front. Cell. Neurosci. 14:18. doi: 10.3389/fncel.2020.00018, PMID: 32116563 PMC7026251

[ref67] Solár P. Zamani A. Kubíčková L. Dubový P. Joukal M. (2020c). Choroid plexus and the blood–cerebrospinal fluid barrier in disease. Fluids Barriers CNS 17:35. doi: 10.1186/s12987-020-00196-2, PMID: 32375819 PMC7201396

[ref68] Solár P. Zamani A. Lakatosová K. Joukal M. (2022). The blood–brain barrier and the neurovascular unit in subarachnoid hemorrhage: molecular events and potential treatments. Fluids Barriers CNS 19:29. doi: 10.1186/s12987-022-00312-4, PMID: 35410231 PMC8996682

[ref69] Solomon R. A. Antunes J. L. Chen R. Y. Bland L. Chien S. (1985). Decrease in cerebral blood flow in rats after experimental subarachnoid hemorrhage: a new animal model. Stroke 16, 58–64. doi: 10.1161/01.str.16.1.58, PMID: 3966267

[ref70] Stridh L. Ek C. J. Wang X. Nilsson H. Mallard C. (2013). Regulation of toll-like receptors in the choroid plexus in the immature brain after systemic inflammatory stimuli. Transl. Stroke Res. 4, 220–227. doi: 10.1007/s12975-012-0248-8, PMID: 23741282 PMC3664758

[ref71] Talsma A. D. Niemi J. P. Pachter J. S. Zigmond R. E. (2022). The primary macrophage chemokine, CCL2, is not necessary after a peripheral nerve injury for macrophage recruitment and activation or for conditioning lesion enhanced peripheral regeneration. J. Neuroinflammation 19:179. doi: 10.1186/s12974-022-02497-9, PMID: 35820932 PMC9277969

[ref72] Thibeault I. Laflamme N. Rivest S. (2001). Regulation of the gene encoding the monocyte chemoattractant protein 1 (MCP-1) in the mouse and rat brain in response to circulating LPS and proinflammatory cytokines. J. Comp. Neurol. 434, 461–477. doi: 10.1002/cne.1187, PMID: 11343293

[ref73] Thompson D. Brissette C. A. Watt J. A. (2022). The choroid plexus and its role in the pathogenesis of neurological infections. Fluids Barriers CNS 19:75. doi: 10.1186/s12987-022-00372-6, PMID: 36088417 PMC9463972

[ref74] Tylek K. Trojan E. Regulska M. Lacivita E. Leopoldo M. Basta-Kaim A. (2021). Formyl peptide receptor 2, as an important target for ligands triggering the inflammatory response regulation: a link to brain pathology. Pharmacol. Rep. 73, 1004–1019. doi: 10.1007/s43440-021-00271-x, PMID: 34105114 PMC8413167

[ref75] Vallières N. Berard J. L. David S. Lacroix S. (2006). Systemic injections of lipopolysaccharide accelerates myelin phagocytosis during Wallerian degeneration in the injured mouse spinal cord. Glia 53, 103–113. doi: 10.1002/glia.20266, PMID: 16206158

[ref76] van Lieshout J. H. Dibué-Adjei M. Cornelius J. F. Slotty P. J. Schneider T. Restin T.. (2018). An introduction to the pathophysiology of aneurysmal subarachnoid hemorrhage. Neurosurg. Rev. 41, 917–930. doi: 10.1007/s10143-017-0827-y, PMID: 28215029

[ref77] Veelken J. A. Laing R. J. Jakubowski J. (1995). The Sheffield model of subarachnoid hemorrhage in rats. Stroke 26, 1279–1284. doi: 10.1161/01.str.26.7.1279, PMID: 7604426

[ref78] Wan Y. Hua Y. Garton H. J. L. Novakovic N. Keep R. F. Xi G. (2019). Activation of epiplexus macrophages in hydrocephalus caused by subarachnoid hemorrhage and thrombin. CNS Neurosci. Ther. 25, 1134–1141. doi: 10.1111/cns.13203, PMID: 31433571 PMC6776740

[ref79] Wei J. Dai S. Pu C. Luo P. Yang Y. Jiang X.. (2022). Protective role of TLR9-induced macrophage/microglia phagocytosis after experimental intracerebral hemorrhage in mice. CNS Neurosci. Ther. 28, 1800–1813. doi: 10.1111/cns.13919, PMID: 35876247 PMC9532915

[ref80] Wei C. Guo S. Liu W. Jin F. Wei B. Fan H.. (2020). Resolvin D1 ameliorates inflammation-mediated blood-brain barrier disruption after subarachnoid hemorrhage in rats by modulating A20 and NLRP3 Inflammasome. Front. Pharmacol. 11:610734. doi: 10.3389/fphar.2020.610734, PMID: 33732145 PMC7957930

[ref81] Wenceslau C. F. Szasz T. McCarthy C. G. Baban B. NeSmith E. Webb R. C. (2016). Mitochondrial N-formyl peptides cause airway contraction and lung neutrophil infiltration via formyl peptide receptor activation. Pulm. Pharmacol. Ther. 37, 49–56. doi: 10.1016/j.pupt.2016.02.005, PMID: 26923940 PMC9731398

[ref82] Wilson E. H. Weninger W. Hunter C. A. (2010). Trafficking of immune cells in the central nervous system. J. Clin. Invest. 120, 1368–1379. doi: 10.1172/JCI41911, PMID: 20440079 PMC2860945

[ref83] Wolburg H. Paulus W. (2010). Choroid plexus: biology and pathology. Acta Neuropathol. 119, 75–88. doi: 10.1007/s00401-009-0627-8 20033190

[ref84] Wu T.-T. Chen T.-L. Chen R.-M. (2009). Lipopolysaccharide triggers macrophage activation of inflammatory cytokine expression, chemotaxis, phagocytosis, and oxidative ability via a toll-like receptor 4-dependent pathway: validated by RNA interference. Toxicol. Lett. 191, 195–202. doi: 10.1016/j.toxlet.2009.08.025, PMID: 19735705

[ref85] Xiang J. Routhe L. J. Andrew Wilkinson D. Hua Y. Moos T. Xi G.. (2017). The choroid plexus as a site of damage in hemorrhagic and ischemic stroke and its role in responding to injury. Fluids Barriers CNS 14:8. doi: 10.1186/s12987-017-0056-3, PMID: 28351417 PMC5371201

[ref86] Yuan J. Lin F. Chen L. Chen W. Pan X. Bai Y.. (2022). Lipoxin A4 regulates M1/M2 macrophage polarization via FPR2-IRF pathway. Inflammopharmacology 30, 487–498. doi: 10.1007/s10787-022-00942-y, PMID: 35235107

[ref87] Zamboni L (1967). Buffered picric acid-formaldehyde: A new rapid fixation for electron microscopy. J. Cell Biol. 35:148A. Available at: https://cir.nii.ac.jp/crid/1571135650533875968 (Accessed July 12, 2022).

[ref88] Zhang Q. Raoof M. Chen Y. Sumi Y. Sursal T. Junger W.. (2010). Circulating mitochondrial DAMPs cause inflammatory responses to injury. Nature 464, 104–107. doi: 10.1038/nature08780, PMID: 20203610 PMC2843437

[ref89] Zhang Z. Zhang A. Liu Y. Hu X. Fang Y. Wang X.. (2022). New mechanisms and targets of subarachnoid hemorrhage: A focus on mitochondria. Curr. Neuropharmacol. 20, 1278–1296. doi: 10.2174/1570159X19666211101103646, PMID: 34720082 PMC9881073

[ref90] Zhao B. N. Campbell J. J. Salanga C. L. Ertl L. S. Wang Y. Yau S.. (2019). CCR2-mediated uptake of constitutively produced CCL2: A mechanism for regulating chemokine levels in the blood. J. Immunol. 203, 3157–3165. doi: 10.4049/jimmunol.1900961, PMID: 31676674 PMC7028331

