# Supplementary material for: Inflammatory changes in the choroid plexus following subarachnoid hemorrhage: the role of innate immune receptors and inflammatory molecules
Source: Front Cell Neurosci. 2025 Jan 7;18:1525415. doi: 10.3389/fncel.2024.1525415 (PMC11747387; doi:10.3389/fncel.2024.1525415)
Supplement: Supplementary file 1 [file Data_Sheet_1.pdf]

Supplementary Material

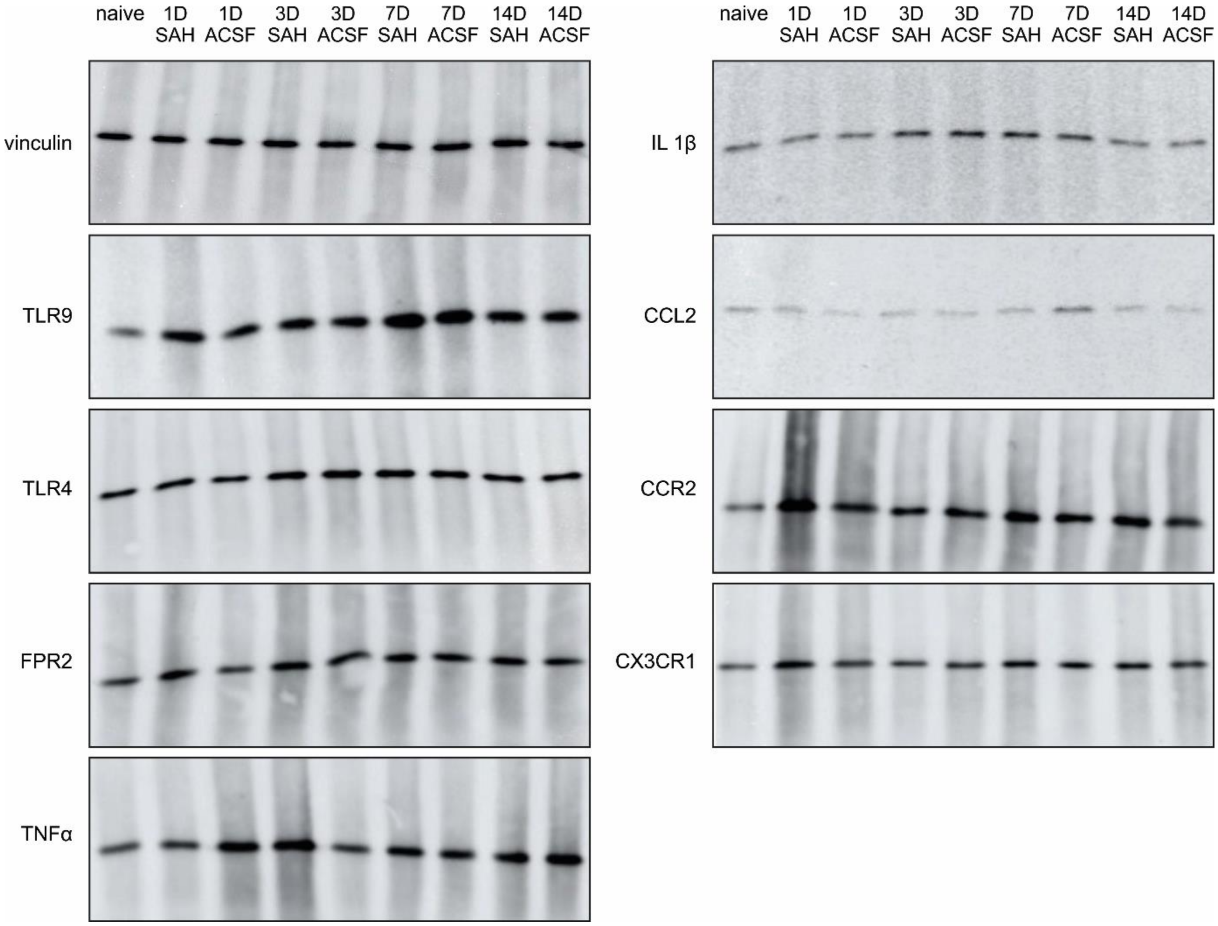

Supplementary Figure 1. Western blot analyses of TLR9, TLR4, FPR2, TNFα, IL-1β, CCL2, CCR2 and CX3CR1 in the CP at different time periods following SAH induction or ACSF application. Vinculin was used as a control. The ECL detection kit (Amersham) was used to visualize protein bands in the LAS-3000 chemiluminometer

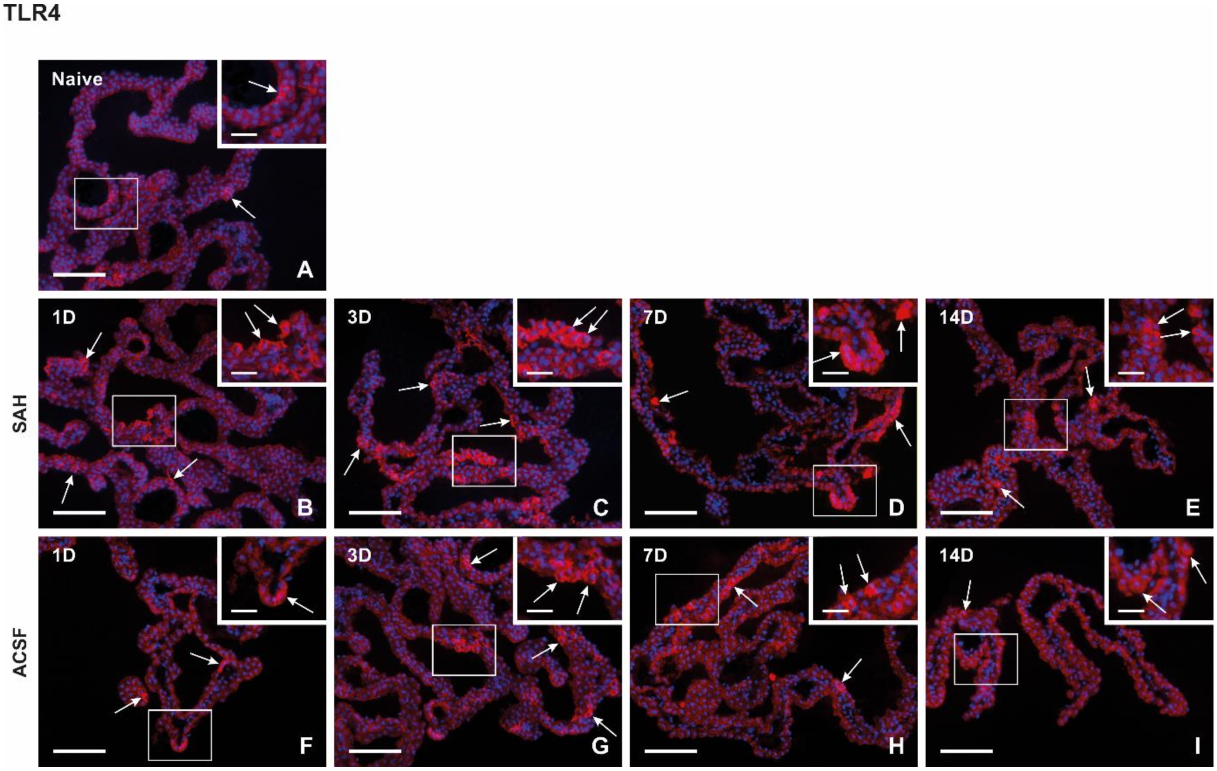

Supplementary Figure 2. Representative pictures showing the results of immunohistochemical staining with TLR4 antibody in cryostat sections of the CP from naïve (A), SAH (B–E), and ACSF (F–I) rats at 1, 3, 7 and 14 days (1D, 3D, 7D and 14D) after SAH induction or ACSF application. Arrows show TLR4 positivity predominantly on the surface of the epithelial cells of the CP. Insets show a higher magnification of regions marked by the boxes. Cell nuclei were detected by staining with Hoechst 33342. Scale bars = 80 µm (main image); 10 µm (insets).

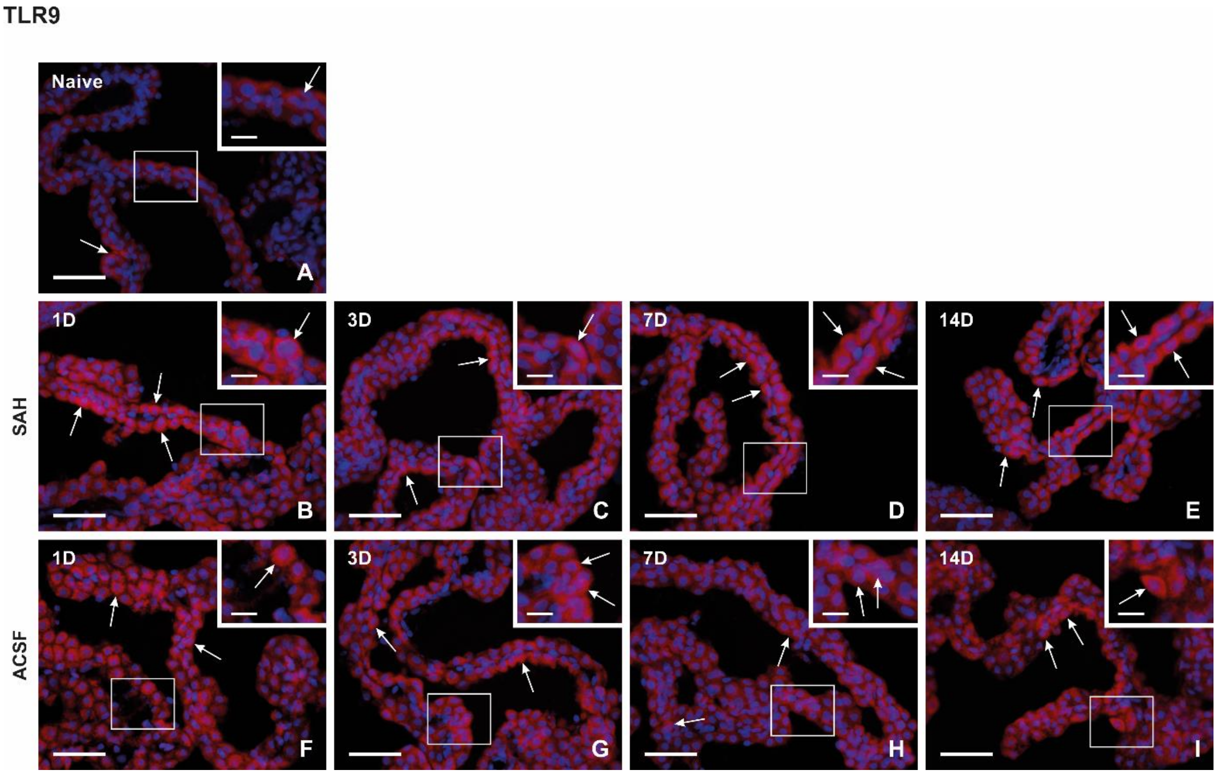

Supplementary Figure 3. Representative pictures showing the results of immunohistochemical staining with TLR9 antibody in cryostat sections of the CP from naïve (A), SAH (B–E), and ACSF (F–I) rats at 1, 3, 7 and 14 days (1D, 3D, 7D and 14D) after SAH induction or ACSF application. Arrows show TLR9 positivity predominantly in CP epithelial cells. Insets show a higher magnification of regions marked by the boxes. Cell nuclei were detected by staining with Hoechst 33342. Scale bars = 80 µm (main image); 10 µm (insets).

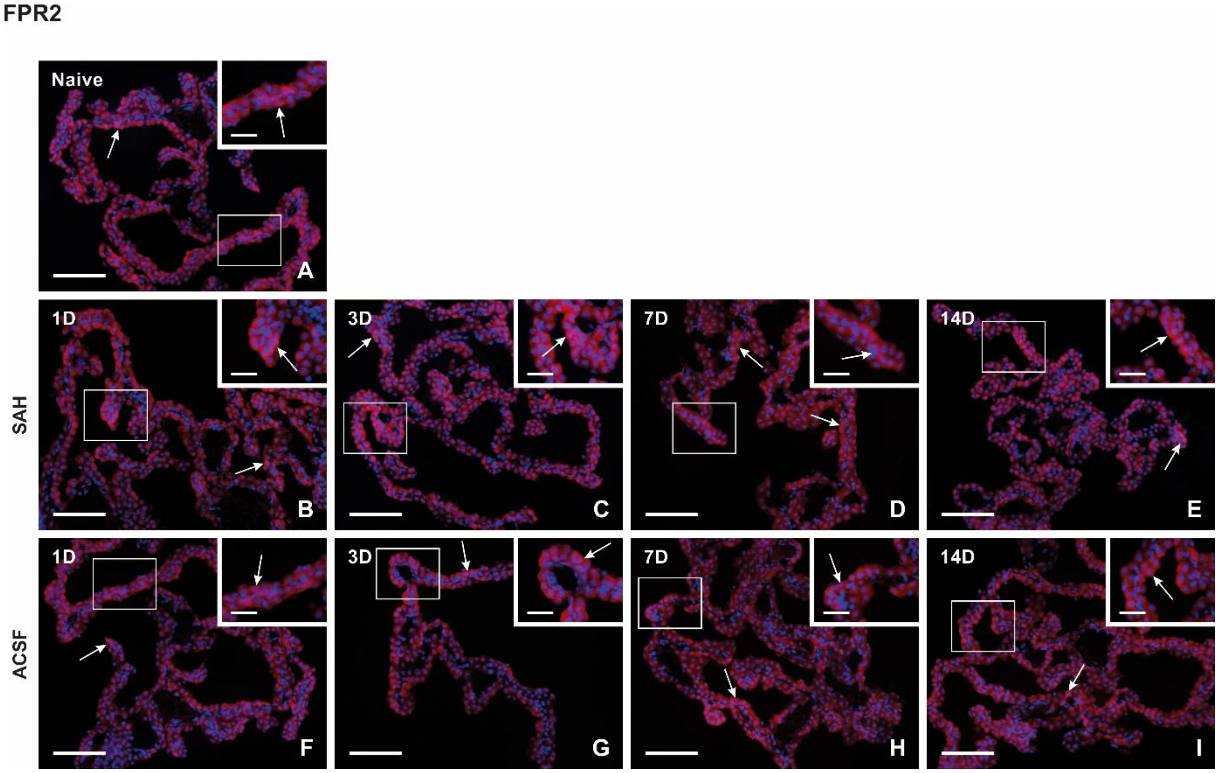

Supplementary Figure 4. Representative pictures showing the results of immunohistochemical staining with FPR2 antibody in cryostat sections of the CP from naïve (A), SAH (B–E), and ACSF (F–I) rats at 1, 3, 7 and 14 days (1D, 3D, 7D and 14D) after SAH induction or ACSF application. Arrows show FPR2 positivity predominantly on the surface of CP epithelial cells. Insets show a higher magnification of regions marked by the boxes. Cell nuclei were detected by staining with Hoechst 33342. Scale bars = 80 μm (main image); 10 μm (insets).

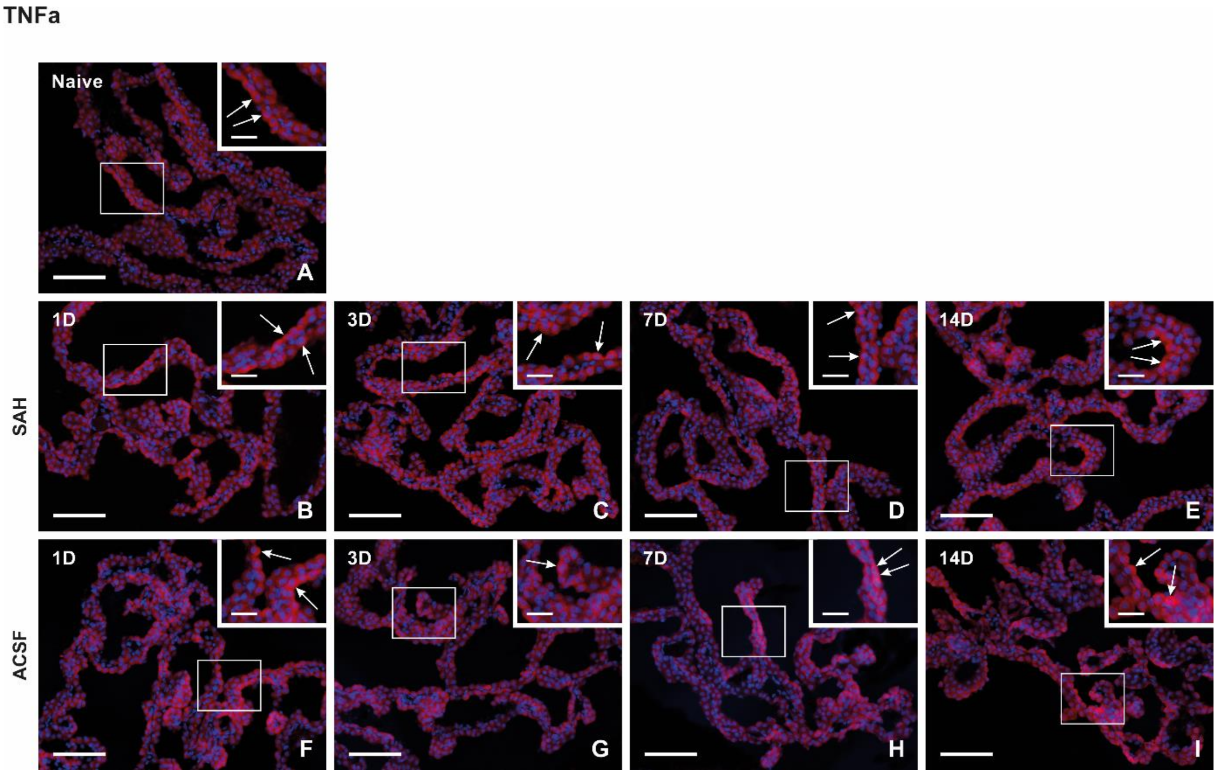

Supplementary Figure 5. Representative pictures showing the results of immunohistochemical staining with TNFα antibody in cryostat sections of the CP from naïve (A), SAH (B–E), and ACSF (F–I) rats at 1, 3, 7 and 14 days (1D, 3D, 7D and 14D) after SAH induction or ACSF application. Arrows show TNFα positivity predominantly in CP epithelial cells. Insets show a higher magnification of regions marked by the boxes. Cell nuclei were detected by staining with Hoechst 33342. Scale bars = 80 µm (main image); 10 µm (insets).

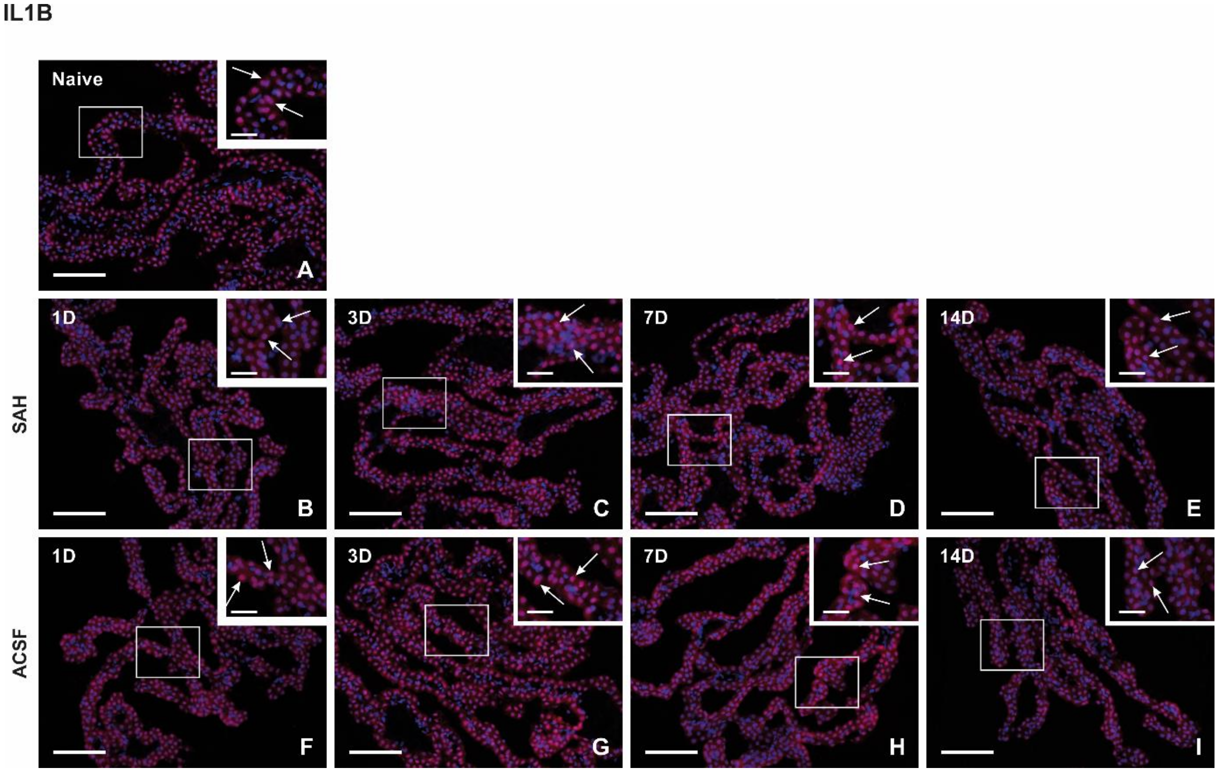

Supplementary Figure 6. Representative pictures showing the results of immunohistochemical staining with IL1β antibody in cryostat sections of the CP from naïve (A), SAH (B–E), and ACSF (F–I) rats at 1, 3, 7 and 14 days (1D, 3D, 7D and 14D) after SAH induction or ACSF application. Arrows show IL1β positivity predominantly in the nuclei of CP epithelial cells. Insets show a higher magnification of regions marked by the boxes. Cell nuclei were detected by staining with Hoechst 33342. Scale bars = 80 µm (main image); 10 µm (insets).

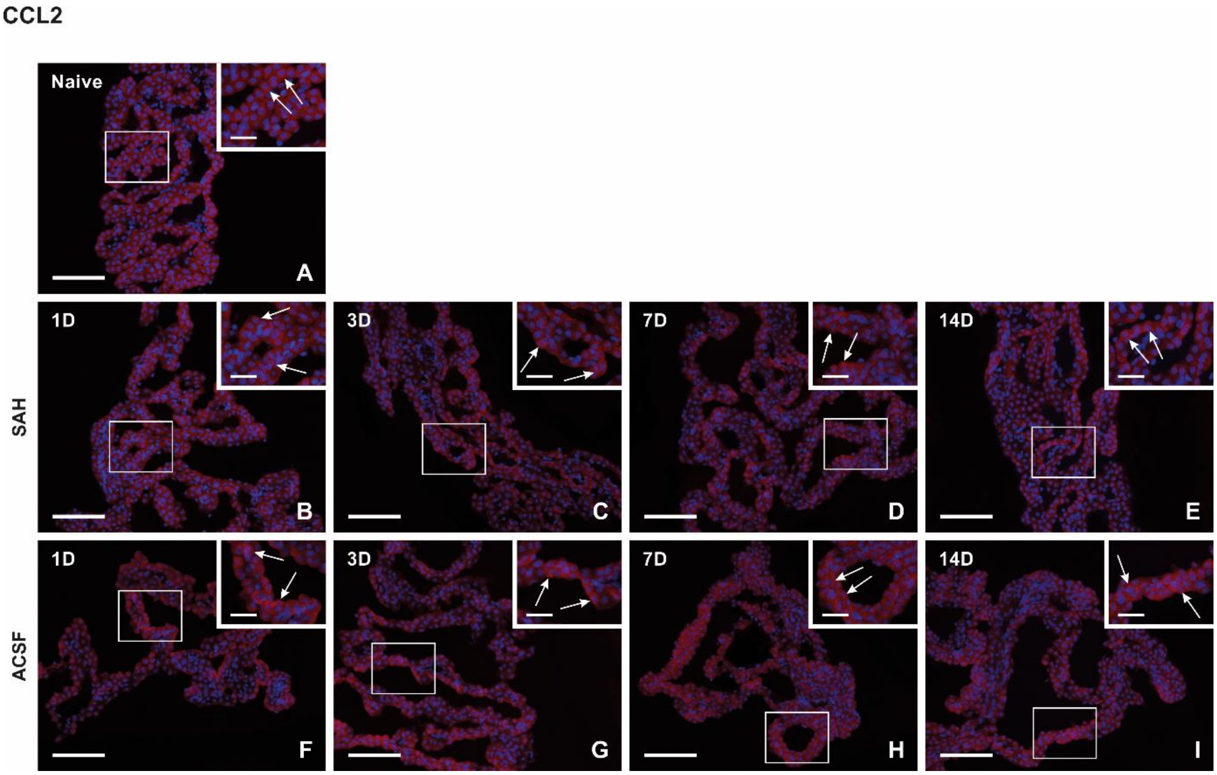

Supplementary Figure 7. Representative pictures showing the results of immunohistochemical staining with CCL2 antibody in cryostat sections of the CP from naïve (A), SAH (B–E), and ACSF (F–I) rats at 1, 3, 7 and 14 days (1D, 3D, 7D and 14D) after SAH induction or ACSF application. Arrows show CCL2 positivity predominantly in CP epithelial cells. Insets show a higher magnification of regions marked by the boxes. Cell nuclei were detected by staining with Hoechst 33342. Scale bars = 80 µm (main image); 10 µm (insets).

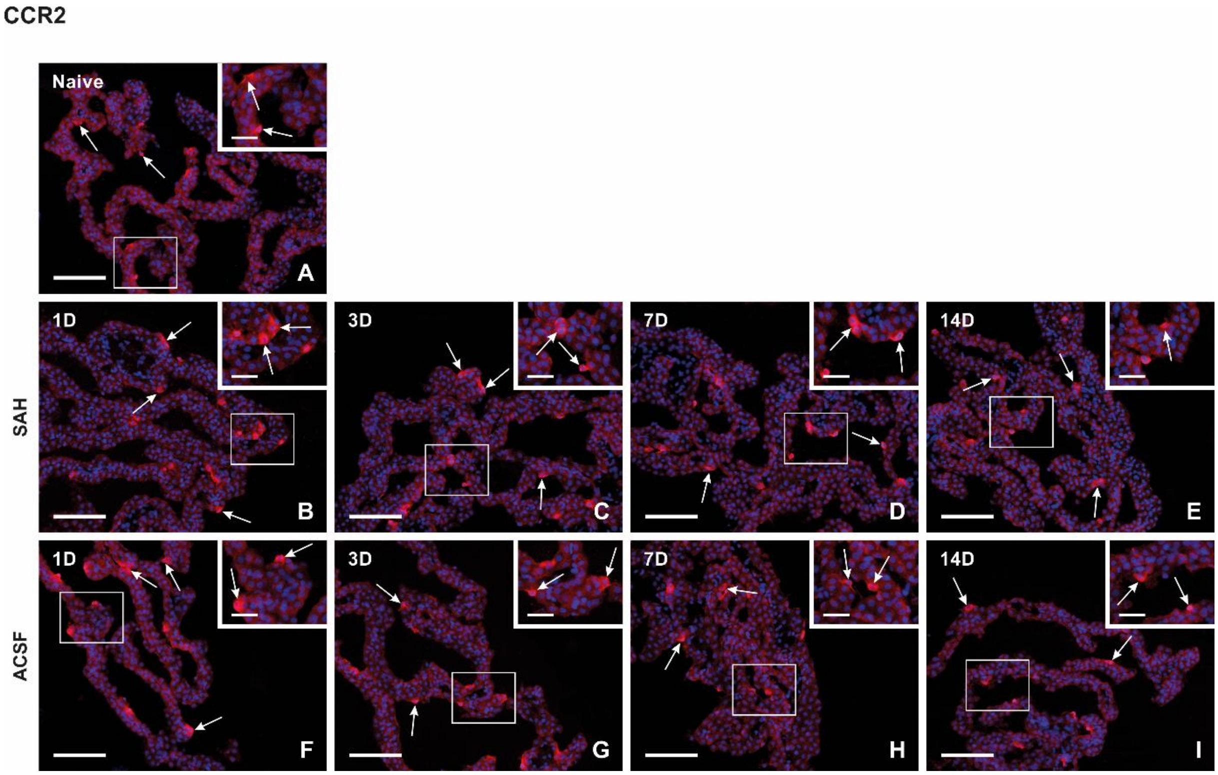

Supplementary Figure 8. Representative pictures showing the results of immunohistochemical staining with CCR2 antibody in cryostat sections of the CP from naïve (A), SAH (B–E), and ACSF (F–I) rats at 1, 3, 7 and 14 days (1D, 3D, 7D and 14D) after SAH induction or ACSF application. Arrows show CCR2 positive cells predominantly in the epiplexus position of the CP. Insets show a higher magnification of regions marked by the boxes. Cell nuclei were detected by staining with Hoechst 33342. Scale bars = 80 µm (main image); 10 µm (insets).

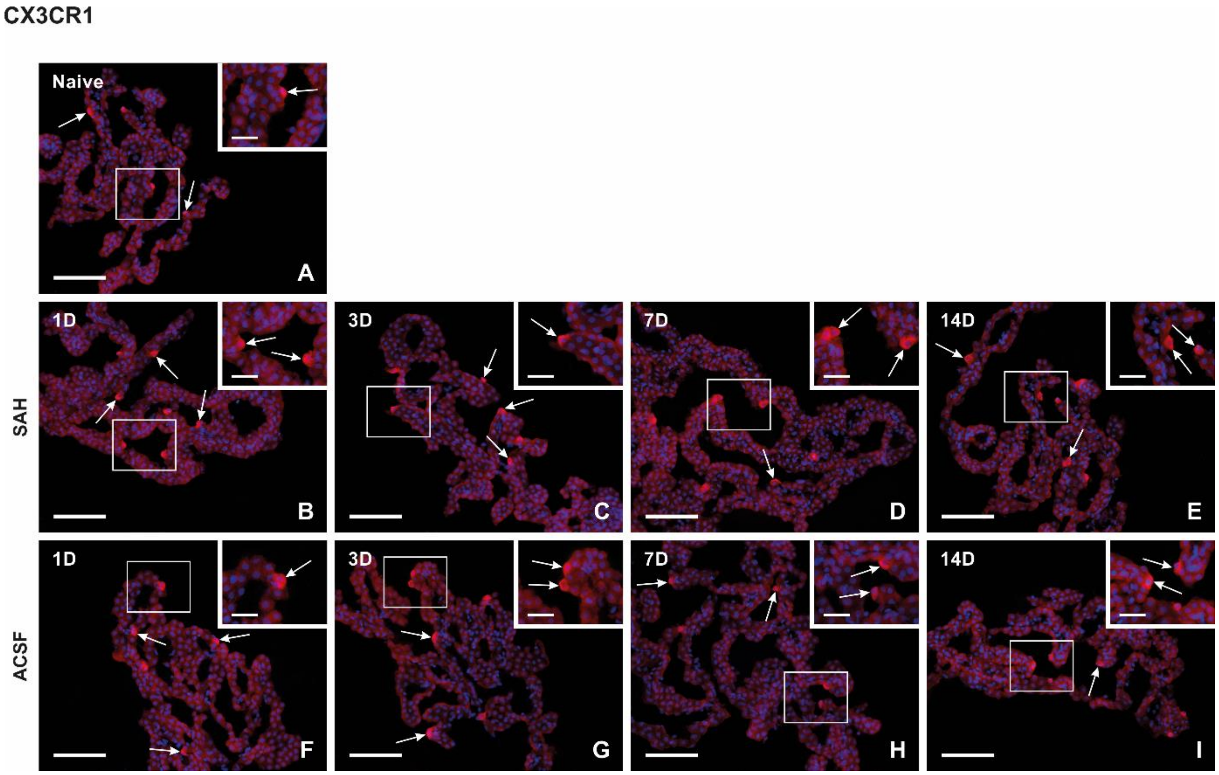

Supplementary Figure 9. Representative pictures showing the results of immunohistochemical staining with CX3CR1 antibody in cryostat sections of the CP from naïve (A), SAH (B–E), and ACSF (F–I) rats at 1, 3, 7 and 14 days (1D, 3D, 7D and 14D) after SAH induction or ACSF application. Arrows show CX3CR1 positive cells predominantly in the epiplexus position of the CP. Insets show a higher magnification of regions marked by the boxes. Cell nuclei were detected by staining with Hoechst 33342. Scale bars = 80 µm (main image); 10 µm (insets).
